# Genomic Basis of Striking Fin Shapes and Colors in the Fighting Fish

**DOI:** 10.1093/molbev/msab110

**Published:** 2021-04-19

**Authors:** Le Wang, Fei Sun, Zi Yi Wan, Baoqing Ye, Yanfei Wen, Huiming Liu, Zituo Yang, Hongyan Pang, Zining Meng, Bin Fan, Yuzer Alfiko, Yubang Shen, Bin Bai, May Shu Qing Lee, Francesc Piferrer, Manfred Schartl, Axel Meyer, Gen Hua Yue

**Affiliations:** 1 Molecular Population Genetics & Breeding Group, Temasek Life Sciences Laboratory, Singapore, Singapore; 2 School of Life Sciences, Sun Yat-sen University, Guangzhou, China; 3 Department of Food and Environmental Engineering, Yangjiang Polytechnic, Yangjiang, China; 4 Biotech Lab, Wilmar International, Jakarta, Indonesia; 5 Key Laboratory of Exploration and Utilization of Aquatic Genetic Resources, Shanghai Ocean University, Shanghai, China; 6 Institute of Marine Sciences (ICM), Spanish National Research Council (CSIC), Barcelona, Spain; 7 Developmental Biochemistry, Biocenter, University of Wuerzburg, Wuerzburg, Germany; 8 The Xiphophorus Genetic Stock Center, Department of Chemistry and Biochemistry, Texas State University, San Marcos, TX, USA; 9 Department of Biology, University of Konstanz, Konstanz, Germany; 10 Department of Biological Sciences, National University of Singapore, Singapore, Singapore; 11 School of Biological Sciences, Nanyang Technological University, Singapore, Singapore

**Keywords:** domestication, evolution, major-effect loci, cis-regulation, mitfa, zic1/zic4

## Abstract

Resolving the genomic basis underlying phenotypic variations is a question of great importance in evolutionary biology. However, understanding how genotypes determine the phenotypes is still challenging. Centuries of artificial selective breeding for beauty and aggression resulted in a plethora of colors, long-fin varieties, and hyper-aggressive behavior in the air-breathing Siamese fighting fish (*Betta splendens*), supplying an excellent system for studying the genomic basis of phenotypic variations. Combining whole-genome sequencing, quantitative trait loci mapping, genome-wide association studies, and genome editing, we investigated the genomic basis of huge morphological variation in fins and striking differences in coloration in the fighting fish. Results revealed that the double tail, elephant ear, albino, and fin spot mutants each were determined by single major-effect loci. The elephant ear phenotype was likely related to differential expression of a potassium ion channel gene, *kcnh8*. The albinotic phenotype was likely linked to a cis-regulatory element acting on the *mitfa* gene and the double-tail mutant was suggested to be caused by a deletion in a *zic1*/*zic4* coenhancer. Our data highlight that major loci and cis-regulatory elements play important roles in bringing about phenotypic innovations and establish Bettas as new powerful model to study the genomic basis of evolved changes.

## Introduction

Already in the “On the Origin of Species” (1859) and later in the “The Variation of Animals and Plants under Domestication” (1868), Charles Darwin recognized that the same processes of selection that act in nature also apply to selective breeding where they are sped up orders of magnitude by breeders’ goals to obtain particular traits. Obviously, Darwin could not know the genomic basis that underlies the selected traits. This began to change, particularly in the last decade, as genome sequences could be obtained for more species. Yet, we are still only at the beginning of understanding how the genotype controls and determines the phenotype (Frazer et al. 2009). Both, mutations in coding sequences and polymorphisms in noncoding sequences are now known to play important roles in generating phenotypic variation (Wittkopp and Kalay 2012; Andersson et al. 2015; Petit et al. 2017; Kemble et al. 2019). Beyond a handful of genetic or developmental model systems (Lehner 2013), organisms under artificial selection not only provide outstandingly useful phenotypes but also permit studying the connection between evolutionary changes and their genomic bases. Knowledge about the bridge between genotypes and phenotypes poses a question, whether the same genomic mechanisms, as Darwin suggested, both in evolution as well as in animal breeding, are at work bringing about innovations (Shapiro et al. 2004; Karlsson et al. 2007; Rubin et al. 2010). Importantly, assays including CRISPR-Cas mediated genome modification that allow establishing functional associations between genotype and phenotype only became recently available.

The Siamese fighting fish (*Betta splendens*), one of the most popular ornamental fishes worldwide, is well-known for its aggressive behavior (Simpson 1968), extremely diverse color patterns, and huge variation in fin shapes (Lucas 1968). It belongs to the anabantoid fishes, characterized by a modified gill skeleton that forms the labyrinth organ, which permits air breathing as these fish tend to live in oxygen-deprived waters. Males build bubble nests and perform complex courting and parental care behaviors (Lucas 1968; Rüber et al. 2004; Monvises et al. 2009). It is a short-lived species and its generation interval is only 5–6 months, with each spawning producing up to several hundred eggs (Monvises et al. 2009). The initiation of domestication of fighting fish has been documented to have occurred as early as 600 years ago, with the purpose of using these fish in staged fighting contests by the Siamese in the current Thailand, leading to the “Plakat” betta (Smith 1945). Selection on other display traits, mainly including coloration and fin shapes, has a more recent origin traced back to the middle of the nineteenth century and was prompted by the use of these fish in exhibition contests (Lucas 1968). In the past decades since these fish became the object of a worldwide aquarium hobby, most artificial selection in fighting fish has focused on modifying the spectacular body colorations and the overgrowth of fins (supplementary fig. S1, Supplementary Material online). Therefore, Siamese fighting fish constitute an unparalleled system for identifying genetic variants underlying both simple and highly complex morphological traits to increase the understanding of the genetic basis of phenotypic variation, between “natural mutants” and domesticated “sports.”

## Results and Discussion

### Genome Assembly and Annotation

We sequenced a homozygous yellow single-tail female and a homozygous transparent double-tail male fighting fish (supplementary fig. S2 and table S1, Supplementary Material online), each with over 120-fold genome coverage. Genome assembly sizes for the female and male were 424.9 and 411.1 Mb, respectively (supplementary note 1, Supplementary Material online). The contig and scaffold N50 sizes for the female were 21.3 kb and 2.1 Mb, respectively, and for the male, 17.3 kb and 1.9 Mb, respectively (supplementary table S2, Supplementary Material online). Both the female and male assemblies showed complete and single-copy BUSCO scores of >95%, indicative of high quality (supplementary table S3 and figs. S3 and S4, Supplementary Material online). A total of 22,977 protein-coding genes were predicted. Transposable elements and other types of repetitive elements together accounted for only 10.7% of the genome sequences (supplementary table S4, Supplementary Material online), a relatively small fraction of the genome compared with other teleosts (Malmstrøm et al. 2017). We also annotated 160,595 conserved noncoding elements (CNEs) and 1,059 lncRNAs in the genome, with a total length of 34.1 Mb (∼8.0%) and 1.47 Mb (∼0.35%), respectively. In comparison to nine other fish genomes, the fighting fish therefore has, besides the pufferfishes, the shortest mean intergenic regions and the lowest overall proportion of noncoding elements within genes, indicating that it has a very compact genome (supplementary fig. S5, Supplementary Material online). Using two high-density linkage maps, 94.9% and 95.3% of scaffolds were anchored on 21 linkage groups corresponding to the chromosomes of the female and male karyotypes, respectively (supplementary tables S5–S7 and fig. S6, Supplementary Material online). Our genome assembly is an important addition to those published recently for the fighting fish (Fan et al. 2018; Prost et al. 2020). Therefore, they supply a useful tool for downstream genetic and genomic studies.

### Genetic Diversity and Population Structure of the Fighting Fish

**Fig. 1.**
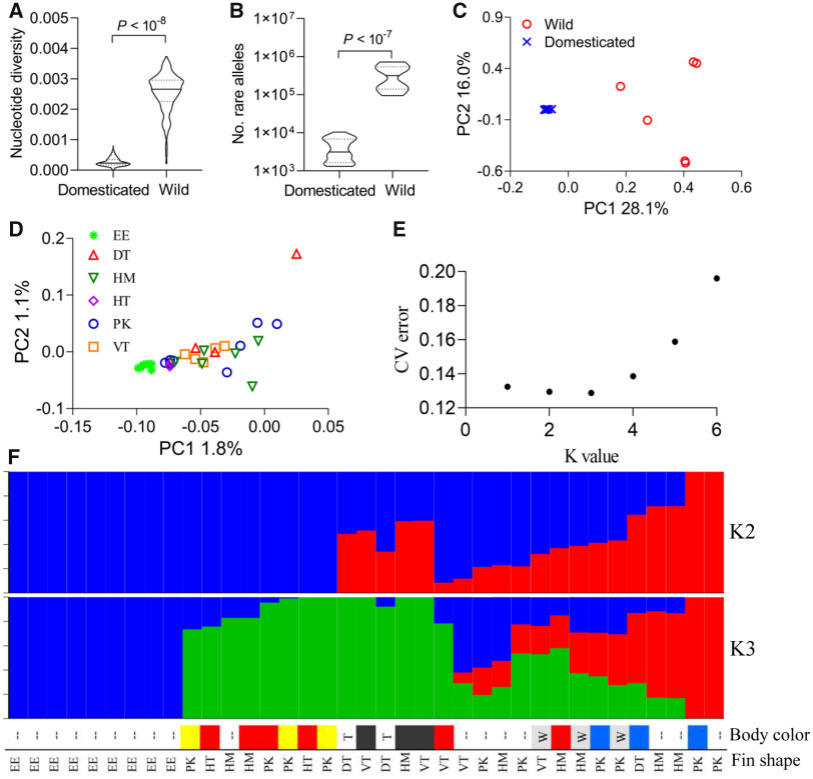
Genetic diversity and population structure in fighting fish. (*A* and *B*) Differences of genetic diversity between domesticated and wild fish measured in nucleotide diversity and number of rare alleles, respectively. *P* values for *t-*test are shown above. (*C* and *D*) Population structure among domesticated and wild fish, and within domesticated fish, respectively, revealed by principal component analysis. EE, elephant ear; DT, double tail; HM, halfmoon tail; HT, horse tail; PK, Plakat tail; VT, veil tail. (*E*) The most likely number of genetic clusters (K) is inferred as 3, where shows the lowest cross-validation errors. (*F*) Population structure at individual level revealed by admixture analysis, at K = 2 and 3, in domesticated fish. Major traits including body color (– indicates too complicated color pattern to phenotype, whereas T and W indicate transparent and white coat color, respectively) and fin shape (codes are corresponding to those in *D*), for each individual, are also shown below.

We sequenced the whole genomes of domesticated fish of diverse coloration and fin traits as well as several wild fish exhibiting ancestral phenotypes (supplementary table S8, Supplementary Material online). Domesticated species tend to lose genetic diversity compared with the wild type. Here, based on whole-genome resequencing data, we observed that the overall genetic diversity in domesticated fighting fish was reduced approximately 10-fold when compared with the fish from the wild (nucleotide diversity: 0.0003 vs. 0.0025, *P *<* *10^−8^ for *t*-test, estimated using VCFtools [Danecek et al. 2011]; unbiased nucleotide diversity: 0.0004 vs. 0.0033, *P *<* *10^−8^ for *t*-test, estimated using pixy [Korunes and Samuk 2021]). Remarkably, the average number of rare single nucleotide polymorphisms (SNPs) with a cutoff value of minor allele frequency of 0.01 was even more decreased, nearly 80 times (4,290 vs. 349,824, *P *<* *10^−7^ for *t*-test; fig. 1*A* and *B*). Such rapid loss of rare alleles during domestication is likely due to genetic bottlenecks during establishment of strains and random genetic drift during domestication, rather than resulting from intense artificial selection imposed by selective breeding (Hyten et al. 2006).

Population structure analyses based on both principal component and admixture analyses consistently showed that the domesticated fish have significantly diverged from the fish from the wild, after several hundred years of selective breeding (fig. 1*C* and supplementary fig. S7, Supplementary Material online). Principal component analysis in the different breeding lines of domesticated fish showed that, except for the elephant ear phenotypes, there was no clear differentiation among the studied traits (fig. 1*D*). In admixture analysis, the most likely number of genetic clusters for hypothesized ancestral groups within domesticated fish was inferred to be three (fig. 1*E*). We observed that fish exhibiting the same particular trait, for example, the halfmoon tail, the double tail, and the Plakat fin shape, are not always assigned to the same genetic clusters (fig. 1*F*). The results imply that these traits are more independent of their genetic background than those determined by a number of minor-effect loci, as in some domesticated animal breeds (Do et al. 2013; Al-Mamun et al. 2015; Wei et al. 2015) and is likely determined by a single or a few loci with major effects.

### Genetic Control of Diverse Pigment Patterns

**Fig. 2.**
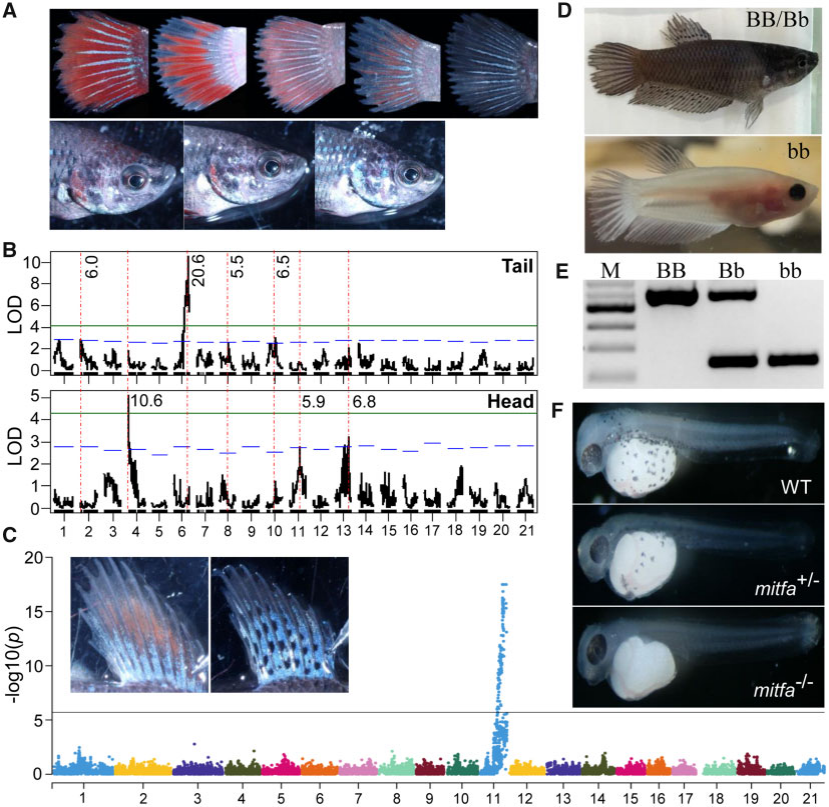
Genetic mapping of distribution of red pigments (xanthophores) and fin-spotting pattern, albino mutant, and validation of *mitfa* gene as the candidate causal gene for albino mutant using CRISPR/Cas9 knockout. (*A*) Variation of the distribution of red pigments in caudal fin and head sections. (*B*) QTL mapping and comparison for distribution of red pigments in caudal fin and head sections, where blue and green horizontal lines indicate LOD cutoff values of chromosome- and genome-wide significance, respectively. PVE (%) by each QTL is shown at the top of each QTL region. Comparisons of QTL distributions between the two traits are indicated with vertical dashed lines. (*C*) Spotted versus nonspotted fin pigmentation patterns in fighting fish and association study using mixed linear modeling, which identified only one locus at LG11 for this trait. (*D* and *E*) The melanin (wild-type pigmented) and albino mutant and their corresponding genotypes based on a deletion flanking *mitfa*. (*F*) The wild-type pigmented fighting fish with regular pattern of melanized cells at 48 hpf (WT), mosaic *mitfa*-knockout fish showing less melanized cells at 48 hpf (*mitfa*^+/−^) and *mitfa*-knockout fish showing no melanized cells throughout the whole embryo at 48 hpf (*mitfa*^-/-^), where no wild-type haplotypes are detected.

The fighting fish is famous for its diversity of striking pigment patterns generated by artificial selection (supplementary fig. S1, Supplementary Material online). First, we examined if pigment-related traits are monogenic or polygenic in several test crosses focusing first on red pigments. We noted that xanthophore density differed markedly between body segments (fig. 2*A* and supplementary fig. S8, Supplementary Material online). Haley–Knott regression quantitative trait loci (QTL) mapping revealed a major locus at LG6 for red pigment distribution in the caudal fin, with 20.6% of its phenotypic variation explained (PVE) by this QTL. Aside from this major QTL, three additional QTLs with significant but smaller effects were identified at LG2, LG8, and LG10, with PVE of 6.0%, 5.5%, and 6.5%, respectively (fig. 2*B*). For pigmentation in the head, we identified one significant and two suggestive QTLs at LG4, LG11, and LG13, with PVE of 10.6%, 5.9%, and 6.8%, respectively (fig. 2*B*). These data show that the distribution of red pigments is a polygenic trait. Interestingly, the QTLs found for tail had no overlap with those for head, implying the distribution of red pigments in different body sections is determined by different genetic loci. Although we did not identify the genes underlying red pigment distribution, our study provides first insights for a better understanding of various pigmentation patterns from a polygenic perspective.

The second pigmentation trait we investigated was dorsal fin spotting (fig. 2*C*). We phenotyped 156 fish from the F_2_ family, RM2, and found that this conforms to a pattern of Mendelian inheritance (supplementary fig. S9*a*, Supplementary Material online). Mixed linear modeling with a genetic relatedness matrix (GRM) based on ∼25 K SNPs revealed only one major locus on LG 11 responsible for this trait (fig. 2*C* and supplementary fig. S9*b*, Supplementary Material online). All fish with dorsal fin spots were homozygous at the most differentiating SNPs, suggesting that this trait is recessive. Using this data set, the locus was restricted to a region of ∼800 kb harboring ∼100 genes (supplementary fig. S9*c*, Supplementary Material online). This genomic region will be the focus for further investigation.

Finally, we studied the albino phenotype, which is characterized by a total lack of black pigments in the fins and body, except for the eyes, regardless of presence of the other colors. This recessive trait follows a monogenic Mendelian inheritance pattern (supplementary fig. S10*a*, Supplementary Material online). We mapped this trait to a locus on LG4 by using RAD-tag markers on our test crosses (supplementary fig. S10*b* and *c*, Supplementary Material online). Recombination analysis based on 293 fish revealed that this locus spans a genomic region of ∼438 kb (supplementary fig. S10*d*, Supplementary Material online), with 18 predicted genes (supplementary S11*a*, Supplementary Material online). Because the albino fish lacked melanin expression in the skin (fig. 2*D* and *E*), we compared the expression pattern of these genes in albino and wild-type pigmented fish and found that only *microphthalmia-associated transcription factor a* (*mitfa*) within this region was differentially expressed (supplementary fig. S11*b*, Supplementary Material online). However, the expression of *mitfa* was also decreased in the eye of the albino fish, which typically shows black pigmentation (supplementary fig. S11*d*, Supplementary Material online). Studying the expression of the *mitfa* gene, we found a paralog of *mitfa*, in the eye. The expression of *mitfb* was higher than that of *mitfa* in the eye of both albino and wild-type fish, and, interestingly, *mitfb* was more highly expressed in eyes of albino than wild-type fish (supplementary fig. S11*d*, Supplementary Material online). It is likely that *mitfb* has critical functions for retinal pigment formation and shows compensatory effects on *mitfa* in albino fish phenotype. Interestingly, this mechanism is consistent with the *nacre* mutant of zebrafish (*Danio rerio*), which is also a mutation of *mitfa* and also has black eyes (Lister et al. 1999, 2001).

To verify whether *mitfa* is associated with the albino phenotype, we knocked out this gene using CRISPR/Cas9 system by targeting the coding sequences (supplementary fig. S12, Supplementary Material online). Two G0 CRISPants without detectable wild-type alleles completely lost melanin pigmentation, whereas the mosaic fish with both wild-type and mutated haplotypes were markedly reduced in the density of melanin-containing cells, compared with wild-type controls, which consistently presented normal melanin pigmentation 48 hpf (fig. 2*F* and supplementary fig. S13, Supplementary Material online). This CRISPRant phenotype matches the phenotype of the albino mutant, suggesting that *mitfa* is the altered gene in this fighting fish mutant. However, we did not find any mutation in introns and exons of *mitfa*, implying that the mutant phenotype is associated with variation in cis-regulatory element acting on this gene. Comparison between homozygous albino and wild-type pigmented fish revealed a cluster of indels and SNPs about 25 kb upstream of *mitfa*, including a 366-bp deletion in the albino mutant. Genotyping this deletion in ∼1,000 fish revealed that this deletion was strictly correlated with the albino phenotype (supplementary fig. S14 and table S9, Supplementary Material online). These data suggest that the 366-bp deletion is a distant cis-regulatory element and could underlie the albino phenotype.

Taken together, in the fighting fish, a single or very few major loci can bring about phenotypic innovations for some colors. Thus, these traits are more easily affected by selection than polygenic traits. Certainly, many of the other color strains in the fighting fish are likely to be determined by major-effect loci. Further studies on these traits will provide valuable information to understand the mechanism of how selection affects phenotypic innovations.

### Genetic Basis of Elephant Ear and Double-Tail Varieties

**Fig. 3.**
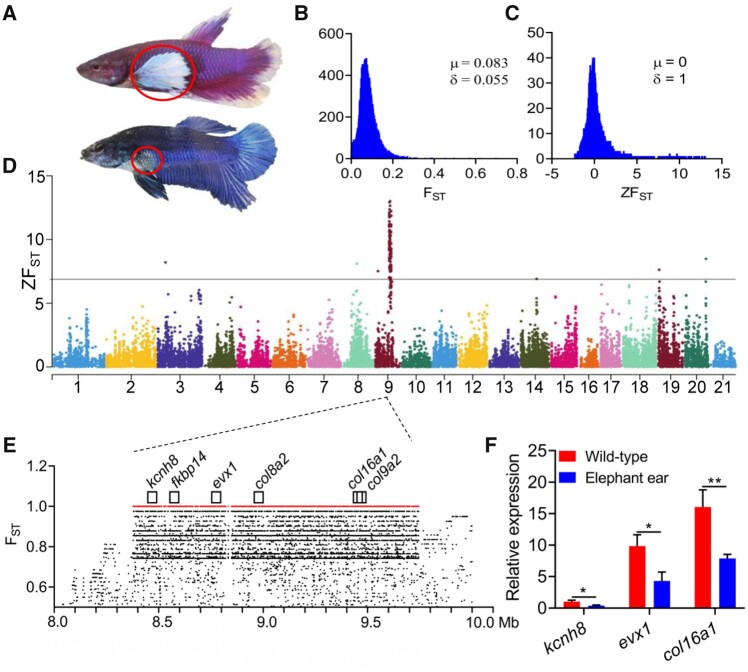
Mapping and identifying candidate genes for elephant ear mutant of fighting fish. (*A*) Elephant ear mutant showing overgrowth of pectoral fin (highlighted with circle), in contrast to wild-type fish. (*B* and *C*) Distribution of F_ST_ and Z-transformed F_ST_ of 30-kb window size for whole-genome-wide variants between elephant ear and wild-type samples, respectively. (*D*) Whole-genome scan identifies a major locus at LG9 for elephant ear using Z-transformed F_ST_. Genome-wide significance cutoff value is denoted with horizontal line. (*E*) Six protein-coding genes associated with fin development and regeneration are predicted in the elephant ear haplotype with a length of ∼1.3 Mb. Fixed variants are denoted with red. (*F*) Three genes including *kcnh8*, *exv1*, and *col16a1* are significantly downregulated in elephant ear mutants (*P <* *0.05, **P <* *0.01; *n* = 3, *t*-test).

Another striking feature of domesticated fighting fish is the overgrowth of almost all types of fins. Using 47 sequenced fish including nine “elephant ear” phenotypes (supplementary table S8 and fig. S1*f*, Supplementary Material online), we firstly mapped the locus for the elephant ear mutation (fig. 3*A*), a recessive trait following Mendelian inheritance characterized by elongated pectoral fins (Lucas 1968). Using *F*_ST_ scans based on whole-genome resequencing data, this locus locates to a 1.3-Mb region on LG9 (fig. 3*B*–*D*). We further refined the haplotypes between elephant ear and wild-type fish and annotated 55 protein-coding genes in this region, of which six are known to play important roles in fin development and regeneration (fig. 3*E*). Examining their expression patterns in pectoral fins at 1 month posthatching when the elephant ear mutation becomes fully apparent, we identified three interesting candidate genes: potassium voltage-gated channel subfamily H member 8 (*kcnh8*), homeobox even-skipped homolog protein 1 (*evx1*), and collagen alpha-1(XVI) chain (*col16a1*) that were significantly downregulated in elephant ear phenotypes when compared with wild-type fish (fig. 3*F*), an observation that agrees with the recessive inheritance pattern of this trait (Lucas 1968). A previous study suggested that *evx1* is required for joint formation in zebrafish fin dermoskeleton, but, apparently has no role in fin length (Schulte et al. 2011). Though important in fin regeneration and typically affected by domestication processes (Anastasiadi and Piferrer 2019), there was no obvious evidence that collagen genes are responsible for overgrowth of fins (Durán et al. 2011; Anastasiadi and Piferrer 2019). In particular, we found one paralog of *kcnh8* at LG14, implying the functions of these two paralogs might have diverged with one fulfilling the general neural function and the other one regulating fin growth, a situation resembling what has been observed regarding the expression patterns of potassium channels in both zebrafish and goldfish (*Carassius auratus*) long-fin mutants (Perathoner et al. 2014; Lanni et al. 2019; Stewart et al. 2019; Kon et al. 2020). Interestingly, in one swordtail species, *Xiphophorus hellerii*, differential expression of *kcnh8* was associated with development of a male ornamental trait, a ventral outgrowth of the caudal fin, called sword (Schartl et al. 2020). In zebrafish and goldfish long-fin mutants, mutations in paralogous potassium channel genes *kcnh2a*, *kcnk5b*, and *kcc4a*, cause overgrowth of different types of fins (Perathoner et al. 2014; Lanni et al. 2019; Stewart et al. 2019; Kon et al. 2020). Mutations disrupting ion channels and ion-dependent signaling often are related to abnormal organ development and regeneration via bioelectrical regulation (McLaughlin and Levin 2018). As discussed above, expression alteration and subfunctionalization of *kcnh8* encoding a potassium voltage-gated channel are more likely related to the formation of the elongated pectoral fins (elephant ear) breed. We found a fixed missense mutation in the last exon (2912 A/G, exon16) of *kcnh8*, but not in the other candidate genes. This amino acid change (H/R) is neither evolutionary conserved across teleosts (supplementary fig. S15, Supplementary Material online) nor predicted to likely affect protein function, with a score of 1.00 as estimated by SIFT (Sim et al. 2012). However, an SNP in coding sequences might be less effective altering gene expression (Cowper-Sal et al. 2012). Except for this SNP, there are still some SNPs and short sequence variations in the noncoding sequences within and closely flanking these candidate genes that may affect expression. Therefore, the elephant ear breed is more likely caused by mutations that affect expression. In addition, it is also worth mentioning that the *FKBP prolyl isomerase 14* (*fkbp14*), encoding a chaperone and calcium-binding protein, shows a similar expression pattern with *kcnh8* and the statistical significance for differential expression is only slightly over 0.05 (fold change, 2.04 and *P* value for *t*-test, 0.07). In zebrafish, inhibition of *fkbp14* function was shown to cause outgrowth of the caudal fin margin (Kujawski et al. 2014). In swordtails, the expression pattern of a paralogous gene *fkbp9* was also observed to be associated with the development of sword of the tail fin in males (Schartl et al. 2020). These data imply that *fkbp14* is another potential candidate gene for “elephant ear” phenotypes. Taken together, our results suggest that a variety of potassium channel and/or calcium-binding genes play critical roles to generate favored ornamental phenotypes of overgrowth of various fin types that are observed in artificial selective breeding in the fighting fish (Stewart et al. 2019; Kon et al. 2020; Schartl et al. 2020).

**Fig. 4.**
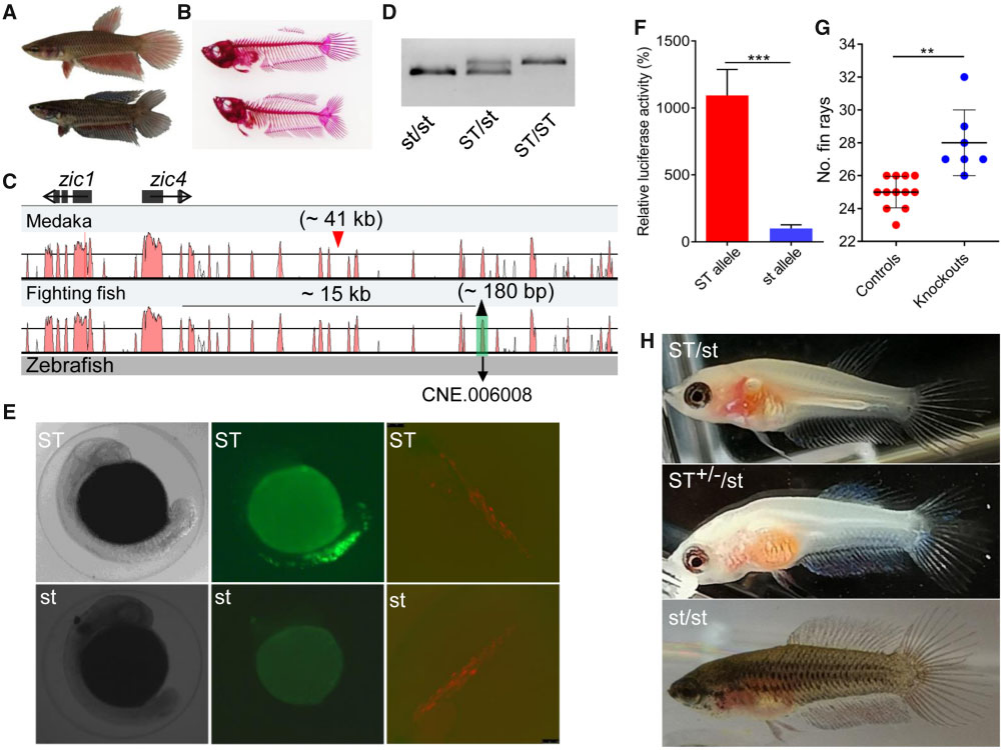
Deletion in the putative enhancer of *zic1* and *zic4* is associated with double-tail mutant. (*A*) Overview of the wild-type (single-tail) and double-tail mutant fighting fish. (*B*) Skeleton staining shows the numbers of fin rays of both dorsal fin and caudal fin are significantly higher in double tail than in single tail. (*C*) Vista plotting of the genomic locus for double-tail mutation among zebrafish, fighting fish, and medaka. Zebrafish is used as reference. Approximately 180-bp deletion located at ∼15-kb downstream of *zic4* is screened overlapping with predicted CNE.006008 of double-tail allele. The insert position of transposon Albatross (∼41 kb) in medaka *Da* locus is indicated with red triangle. (*D*) PCR screening of the deletion in single-tail and double-tail fish. (*E*) Representative fighting fish injected with enhancer detection vector ZED constructed with CNE.006008 from single-tail allele (ST) showing GFP expression predominantly in the dorsal fin and caudal fin positions, and those injected with double-tail allele (st) showing no GFP expression in the whole embryos at 24 hpf. RFP that is only detectable, particularly in muscles, since 72 hpf, is used as internal control. (*F*) Relative luciferase activity in Singapore grouper embryonic cell line transfected with pGL3-Promoter constructs including CNE.006008 region separately from the single-tail and double-tail alleles (Mann–Whitney *U* test, *** *P *<* *0.001). (*G*) The total number of fin rays of dorsal and caudal fins between genetically modified fish (*n* = 7) and its corresponding controls (*n* = 12) in CNE.006008 (Mann–Whitney *U* test, ** *P *<* *0.01). (*H*) The knockout fighting fish (ST^+/−^/st), with ∼60% of ST allele sequences deleted at CNE.006008, shows much more fin rays both in dorsal fin and caudal fin than the single-tail (ST/st) control, but less than double-tail control (st/st). Heterozygous ST/st fish were used as recipients for the CRISPR/Cas9 injections.

Double tail is one of the most well-known and most appreciated among various fin varieties of fighting fish. This mutant presents a unique ventralized pattern of dorsal trunk and tail, and features a doubling of the number of fin rays for both dorsal and caudal fins (fig. 4*A* and *B* and supplementary fig. S16, Supplementary Material online). Double-tail fighting fish was found to be a recessive homozygote (*st*) and we mapped the locus responsible for double tail to a ∼130-kb region on LG1 (*st* vs. *ST*) by RAD sequencing and fine mapping by examination of recombinants in 502 fish (supplementary fig. S17, Supplementary Material online). Sequence analysis revealed that this locus harbors three genes: zinc finger transcription factors Zic1 and Zic4 (*zic1*and *zic4*) and phospholipid scramblase 1 (*plscr1*), and overlaps with the *Da* locus of medaka (*Oryzias latipes*) for a double-tail mutant that contains only *zic1* and *zic4* (Moriyama et al. 2012). Consistent with medaka, expressions of both *zic1* and *zic4* were suppressed in double tail (supplementary fig. S18, Supplementary Material online) and no mutation was identified in the coding sequences (Kawanishi et al. 2013). We further sequenced the genomes of both homozygous single- and double-tail fish and found in double tail no large sequence variation except for a ∼180-bp deletion ∼15-kb downstream of *zic4* (fig. 4*C* and supplementary fig. S19*a*, Supplementary Material online). This deletion was located in a cluster of CNEs and coincided with predicted CNE.006008 (supplementary fig. S19*b*, Supplementary Material online). Genotyping at this locus showed that the deletion was completely correlated with phenotypes in > 1000 examined fish (fig. 4*D* and supplementary table S10, Supplementary Material online). In medaka, both genes, *zic1* and *zic4*, were verified to be responsible for double tail (Moriyama et al. 2012). However, the mechanism by which these genes induce this phenotype is still unclear. It was assumed that a transposon, *Albatross* (> 41 kb), inserted into the common regulatory region of both *zic1* and *zic4*, ultimately leads to the double-tail mutation of medaka (Moriyama et al. 2012). Therefore, we hypothesized that the deletion in CNE.006008, an enhancer, is responsible for the double-tail phenotype in fighting fish. To test this hypothesis, first we inserted the CNE.006008 locus and its closely flanking sequences of ∼100 bp separately from single- and double-tail fish into Zebrafish Enhancer Detection (ZED) vectors (Bessa et al. 2009) and injected them into one-cell stage embryos. We observed that the wild-type *ST* allele significantly enhanced green fluorescent protein (GFP) expression in embryos at 24 hpf, when both *zic1* and *zic4* show differential expression between double-tail and wild-type fish (Moriyama et al. 2012), whereas no visible GFP expression was detected for the *st* allele (fig. 4*E* and supplementary table S11, Supplementary Material online). The efficiency of the two alleles as candidate enhancers was further examined using a Dual-Luciferase Reporter Assay, which showed that the *ST* allele enhanced luciferase expression by ∼10× relative to *st* allele in Singapore grouper embryonic cell line (fig. 4*F*).

Finally, we deleted this enhancer using the CRISPR-Cas9 system in fighting fish. Considering the efficiency of tested gRNAs and the cluster of CNEs that could have unpredicted functions, we limited the modification to the CNE.006008 region and did not involve the other CNEs (supplementary fig. S20, Supplementary Material online). Genetic analysis revealed that none of these fish had completely deleted CNE.006008, suggesting nonsimultaneous cutting at multiple targeted gRNA positions. These mosaic fish (*n* = 7) had significantly more fin rays than the noninjected controls (*P *<* *0.01; fig. 4*G*). We screened one modified fish, where >80% of sequenced clones were mutants with deleted sequences up to 56% of *st* allele (supplementary fig. S21, Supplementary Material online). Although this fish was not a pure knockout, we observed that the number of fin rays of both dorsal and caudal fins was significantly higher than in the single tail and approaching that of the double tail (fig. 4*H*). Taken together, deletion of the candidate coenhancer of *zic1* and *zic4*, CNE.006008 was found to be the causative mutation for double-tail fighting fish.

To date, double-tail mutants are only reported in the fighting fish and medaka, and both are caused by mutations in the coregulatory regions of *zic1* and *zic4* (Moriyama et al. 2012). As shown in medaka loss of function of either gene is not able to cause a double-tail phenotype (Moriyama et al. 2012). Furthermore, loss of functions of both *zic1* and *zic4* causes fatal Dandy–Walker malformation-like disease in animals (Grinberg et al. 2004; Blank et al. 2011). Thus, selection on the coregulatory regions of multiple effector genes in a single locus becomes more efficient to bring about such phenotypic innovations. The occurrences of those kinds of mutations are scarcer than those determined by a single gene, which likely explains why only two double-tail cases in a number of domesticated teleosts have been observed so far. In comparison, traits that are determined by single genes are much more common. Microphthalmia-associated transcription factors have been extensively reported responsible for the albinism of a number of domesticated animals, such as dogs (Karlsson et al. 2007), pigs (Chen et al. 2016), ducks (Zhou et al. 2018), and quails (Minvielle et al. 2010). Interestingly, most of them are caused by mutations in the regulatory sequences (Karlsson et al. 2007; Hauswirth et al. 2012; Chen et al. 2016; Zhou et al. 2018; Hofstetter et al. 2019). Mutations in coding sequences are more likely to alter protein functions. In particular for pleiotropic factors, such mutations will be more harmful to the organisms than those occurring in regulatory regions, which only affect the expression level (Wittkopp and Kalay 2012; Petit et al. 2017). In this regard, mutations in the coding sequences of microphthalmia-associated transcription factors have been extensively reported to lead to various defects in animals (Tassabehji et al. 1994; Levy et al. 2006). This type of mutation is more prone to be eliminated by artificial selection. Above all, mutations in cis-regulatory elements provide valuable raw materials for selection during domestication and play critically important roles in phenotypic variation.

## Conclusion

In this study, we sequenced the genomes of several fighting fish breeds and studied the genomic basis of most striking color and fin shape variants in this species. We found that phenotypes including some colorations and fin shapes were determined by major-effect loci, indicating that major loci can bring about phenotypic innovations rapidly. Using CRISPR/Cas-9 induced-mutations, we verified that both double-tail and albino phenotypes resulted from mutations in a regulatory element near *zic1*/*zic4* and mutations in coding regions of *mitfa*, respectively. Our findings suggest that cis-regulatory elements play critically important roles in generating phenotypic variation during domestication by artificial selection as well as natural selection at evolutionary time scales. There are still other breeds varying in fin shapes and sizes as well as in pigment patterns and in aggression worthy of further investigation by the CRISPR/Cas9 methods we developed here. This will facilitate that this species will become a new model system since it is amenable for dissecting the genetic architecture underlying morphological and behavioral evolutionary innovations.

## Materials and Methods

### Genome Sequencing and Assembly

Genomic DNA from one highly inbred yellow single-tail female and one transparent double-tail male were used for construction of both short-insert (∼270, 350, and 550 bp) and jumping libraries (3, 5, 10, 15, and 20 kb) (supplementary table S1 and fig. S2, Supplementary Material online). In addition, one female (albino and double-tail) and one male (also homozygous for melanin pigmentation and single-tail) were sequenced with 550-bp insert libraries. Genomic DNA was isolated with MagAttract HMW DNA Kit (Qiagen). Heterozygosity versus homozygosity of these fish was assessed at ten microsatellite loci development in a previous study (Chailertrit et al. 2014). The yellow female showed a low genetic heterozygosity of 0.1. Genotypes of these fish were determined by both test crosses and genetic markers developed as described below. All sequencing was carried out on Illumina Nextseq 500. Raw reads were cleaned using the program *process_shortreads* (-r -c -q -t 150) in the Stacks software package (Catchen et al. 2013). Genomes of the highly inbred yellow single-tail female and the transparent double-tail male of varying libraries were assembled using ALLPATHS-LG (Gnerre et al. 2011) with default parameters, whereas the other two unique libraries were assembled using ABYSS2.0 (Jackman et al. 2017) with default parameters. Gaps were filled with paired reads using GapFiller (Nadalin et al. 2012). The genome size of the fighting fish was estimated based on k-mer frequencies, de novo RADtags mapping and Q-PCR method (supplementary note 1, Supplementary Material online). Completeness of the genome assembly was evaluated using BUSCO (Simão et al. 2015) and by the mapping rate of transcripts and de novo RADtags.

### RNA Sequencing and Analysis

Three mRNA libraries were separately constructed for one male and two females of 3 months’ age. Total RNA was isolated from brain, eye, skin, gill, muscle, intestine, spleen, liver, heart, kidney, and gonad, and then equal amounts from each tissue were pooled for library construction using Illumina TruSeq RNA sample preparation kit (Illumina). Moreover, RNA samples of another mature male and female, derived from the above pooling strategy, were used for total RNA library construction with rRNA depletion, using NEBNext Ultra RNA Library Prep Kit (NEB). Raw sequences were cleaned with *process_shortreads* (-r -c -q -t 150) in Stacks package (Catchen et al. 2013). Transcripts of individuals were assembled using Trinity (Haas et al. 2013) with default parameters and then used for genome annotation.

### RADseq and SNP Genotyping

Samples from both mapping families and cultured strains with specific traits (see below), were genotyped using RADseq (Baird et al. 2008) with some modifications as described in our previous study (Bai et al. 2018). High-quality genomic DNA of 500 ng was digested with restriction enzyme *PstI-HF* (NEB) and ligated to barcoded adaptors with T4 DNA Ligase (NEB). DNA was then sheared with a peak of 500 bp for library construction. All libraries were sent to NextSeq500 (Illumina) for 150-bp single-end sequencing. Parental and offspring samples were sequenced with an average of 16.9 and 6.1 M reads, respectively, for accurate SNP calling (supplementary table S1, Supplementary Material online). Raw reads were filtered using *process_radtags* (-r -c -q -t 150) in Stacks package (Catchen et al. 2013). BWA-MEM (Bessa et al. 2009) was used for reference-based mapping with default parameters and only reads with unique targets were retained. SNPs were discovered and genotyped using Stacks package (Catchen et al. 2013) with parameters as described in our previous study (Bai et al. 2018).

### Linkage Mapping and Chromosomal-Level Genome Assembly

Two F_2_ families: BM1 and RM2 were used for linkage mapping. These two F_2_ families: BM1 (92 fish) and RM2 (274 fish) were generated with two pairs of F_1_ parents (i.e., BM1female × BM1male and RM2female × RM2male), respectively, which were the offspring of P parents: DtY2female and F0B1male (see details about their phenotypes in the subsection “Genetic mapping for traits of interest”). Genotyping of the two F_2_ mapping families was conducted using RAD sequencing as described above. SNPs were firstly filtered for Mendelian segregation distortion using χ^2^ tests (*P *<* *0.05). The cutoff value of missing genotypes across families was <15%, which left 80 and 213 samples for BM1 and RM2, respectively, for linkage mapping. Linkage group assignment and marker ordering were carried out using Lep-MAP3 (Rastas 2017) with logarithm of the odds (LOD) cutoff of 10. Both sex-averaged and sex-specific maps were constructed. The constructed linkage maps (Supplementary linkage maps, Supplementary Material online) were used to build a chromosome-level genome assembly. RAD sequences of mapped markers were aligned to scaffolds to examine the occurrence of chimeric assemblies using ALLMAPS (Tang et al. 2015), as linkage maps are not likely to generate among-chromosome grouping errors (Small et al. 2016). If there were more than three markers from the same scaffolds mapped to different linkage groups, the scaffolds were split at the longest gaps between mismatched fragments. The new scaffolds were then anchored onto genetic maps to generate chromosome-level assemblies using ALLMAPS (Tang et al. 2015) with default parameters.

### Genome Annotation

Annotation of the highly inbreed female fighting fish genome was conducted using MAKER (Cantarel et al. 2008). The sequences were softmasked using RepeatMasker (Chen 2004) based on the repeat libraries obtained from RepeatModeler (http://www.repeatmasker.org), Repbase (Jurka et al. 2005), and MAKER (Cantarel et al. 2008) sequence repeat databases. Both evidence-based and ab initio gene models were used for annotation. Transcriptomes of fighting fish and protein sequences of zebrafish, medaka, stickleback, fugu, and Nile tilapia from Ensembl database (release 86) were used for evidence. SNAP (Korf 2004) and Augustus (Stanke and Waack 2003) were iteratively used for ab initio gene models training. Predicted protein sequences were annotated by blast to nr and RefSeq databases (Pruitt et al. 2005) with BLASTP (*E*-value < 1E−10).

### Prediction of CNEs

Identification of CNEs was according to a previous method (Brawand et al. 2014). In brief, the fighting fish genome was used as reference for pairwise whole-genome alignment with zebrafish (*D. rerio*), medaka (*O. latipes*), stickleback (*G. aculeatus*), fugu (*T. rubripes*), and Nile tilapia (*O. niloticus*) (downloaded from Ensembl database, release 86) using LASTZ (Harris 2007). Multiple alignments were generated with MULTIZ (Blanchette et al. 2004) using the tree topology among the six species based on the phylogenetic study. Conserved sequences at least in one pair of alignments were predicted using PhastCons (Siepel et al. 2005) under both conserved and nonconserved models (coverage = 0.3 and length = 45 bp). The predicted CNEs were then filtered by comparison to the coding sequences, noncoding RNAs, pseudogenes, and transposable elements of the six studied species and also the transcripts of fighting fish (*E*-value < 1E^−10^). Only the elements of > 30 bp and with repetitive content <50% were retained. For studies on CNEs related to candidate genes, we manually aligned the genomic loci with more reference fish including nonmodel species to refine the CNEs and identify candidate regulatory elements that might be lineage- or species-specific, using the above standards.

### Whole-Genome Resequencing and Genetic Diversity Analysis

Six wild (four from Thailand and two from Cambodia, respectively) and 28 randomly selected domesticated fish were sequenced with 500-bp insert libraries (supplementary table S8, Supplementary Material online). Nine “elephant ear” mutants, that is, with phenotype of overgrowth of pectoral fin, were further sequenced to identify the genetic locus (supplementary table S8, Supplementary Material online). Raw sequencing reads were filtered using the above method. Sequence mapping and variant calling were carried out using BWA-mem (Bessa et al. 2009) and Picard/GATK v4.0 best practices workflows (DePristo et al. 2011). SNPs were filtered with the following parameters: “QD < 2.0 ‖ FS > 60.0 ‖ MQ < 40.0 ‖ MQRankSum < −12.5 ‖ ReadPosRankSum < −8.0 ‖ SOR > 4.0,” and indels with “QD < 2.0 ‖ FS > 200.0 ‖ ReadPosRankSum < −20.0 ‖ SOR > 10.0.” We further filtered the variants with “minDP 7, –max-missing 0.925” using VCFtools (Danecek et al. 2011). A total of 6,735,573 genotypes were obtained for further analysis. Genetic diversity was estimated with a 100-kb window size using VCFtools (Danecek et al. 2011). Unbiased estimation of nucleotide diversity taking into consideration both segregating and nonsegregating sites were computed using the program pixy (Korunes and Samuk 2021). Population structure was firstly analyzed with principal component analysis (PCA) using Plink2.0 (Purcell et al. 2007). The program Admixture (Alexander et al. 2009) was then used to infer the genetic clusters at individual level.

### Genetic Mapping for Traits of Interest

Three pigmentation traits, including distribution of red pigments in different body compartments (Fig. 2A), dorsal fin spotting (fig. 2C), and albino phenotype (fig. 2D), and two fin morphology traits (i.e., elephant ear and double tail, figs. 3*A*and 4*A*) were studied. Before setting up mapping families by crossing parents with different phenotypes, test crosses were generated to examine phenotypic segregation of double tail, albino, and dorsal fin spotting. Parents that were homozygous for the two studied traits (i.e., double-tail vs. single-tail and melanin vs. albino) were selected as P (parental) generation (DtY2female and F0B1male) to set up mapping families (supplementary fig. S2, Supplementary Material online). Two F_2_ families: BM1 and RM2 were produced by crossing F_1_ parents (BM1female and BM1male) and (RM2female and RM2male), respectively, which were the offspring of P generation: DtY2female and F0B1male. The two F_2_ mapping families were genotyped using RAD sequencing as described above.

In the two F_2_ families (i.e., BM1 containing 92 fish and RM2 containing 274 fish), four traits: distribution of red pigment, dorsal fin spotting, albino, and double tail were recorded for each individual. In detail, F0B1male was a homozygous single-tail and melanin pigmented fish (wild type at both loci), whereas DtY2female was a homozygous double-tail and albino (loss of melanin) fish. Both traits (i.e., double-tail and albino) showed a recessive Mendelian inheritance pattern with a segregation ratio of 3:1 in F_2_ populations. Mixed linear model association analyses were separately conducted based on the two F_2_ families to map the two traits. To narrow down the genomic regions responsible for the two traits, 136 additional fish of confirmed phenotypes, from China, Thailand, Malaysia, Singapore, and Indonesia were genotyped using both RAD sequencing and SNP/indel markers developed from resequencing data, as described above. Genotypes were used to identify recombinants in the major loci determining double-tail and albino. For dorsal fin spotting, parents, DtY2female and F0B1male showed spotted and nonspotted dorsal fin, respectively, whereas F_1_ parents, RM2female and RM2male showed spotted and nonspotted dorsal fin, respectively. Due to a phenotypic interaction of iridescent pigmentation patterns and the albino condition, which also segregated in the F_2_ mapping crosses, only 156 individuals from the RM2 family could be phenotyped. This trait presented a segregation ratio of 1:1 for spotted versus nonspotted dorsal fin. We carried out both quantitative trait loci (QTL) mapping and mixed linear modeling for this trait using these 156 phenotyped fish. As the results in QTL mapping (peak LOD score and PVE were 282.79% and 100%, respectively) and mixed linear modeling were consistent, we only present the results of the mixed linear modeling. Finally, for distribution of red pigment, we observed red pigments in DtY2female, but not in F0B1male, whereas all F_1_ fish presented red pigments. In both F_2_ families, we observed the distribution pattern of red pigments varied evidently not only across different body compartments of each individual but also among individuals throughout the whole family. We developed a method to quantify and record red pigments in different body areas (supplementary note 2, Supplementary Material online). QTL mapping was conducted in the large RM2 family with 211 phenotyped offspring, rather than BM1 with only 77 phenotyped siblings, to map and estimate the effects of the loci.

Mixed linear modeling was performed with compressed mixed linear model implemented in the GAPIT R package, with the GRM and sex as covariates (Lipka et al. 2012). This mixed model incorporates and estimates component variance of both kinship relatedness matrix and sex, using VanRaden algorithm. *P* value was calculated for each marker and the statistical significance threshold was determined at 0.05 level with Bonferroni corrections (*P *=* *0.05/N, where N is the number of total markers used for association test). QTL mapping was conducted using the Haley–Knott regression method (Haley and Knott 1992) implemented in the R package qtl (Broman et al. 2003). The linkage map of RM2 family was used for QTL mapping, with interval mapping algorithm. LOD thresholds for both chromosome- and genome-wide significance were estimated with permutation tests for 1,000 times. To screen the “elephant ear” locus, a genome-wide F_ST_ scan was performed between elephant ear mutants and the remaining resequenced samples, 9 and 28 fish respectively, using 30-kb window size with a step of 15 kb. Window-size F_ST_ values were then Z transformed (ZF_ST_ = (F_ST_ − µF_ST_)/σF_ST_) to compare among chromosomes. Variants within and flanking this locus were retrieved and analyzed to refine the haplotypes. Fixed or nearly fixed variants were annotated and protein-coding genes within this locus were individually analyzed by literature mining. Genes associated with fin development and regeneration were kept for expression analysis using real-time reverse transcription PCR (RT-PCR) to identify candidate genes.

### Developing and Validating Trait-Associated DNA Markers

In order to quickly differentiate among genotypes, we developed indel markers for fast PCR assays for double-tail and albino traits. The shortest genomic region resulting from association mapping was used for marker screening. Homozygous individuals with regard to the above traits were resequenced for marker discovery using GATK pipeline (DePristo et al. 2011) according to our previous study (Wang et al. 2015). Indels were firstly validated by manual alignment of genome sequences between homozygous mutant and wild type. Primers of these indels of suitable length were then designed for PCR assays. The associations between phenotypes and these discovered markers were further tested in domesticated fish from different strains (from ∼500 to ∼1000 individuals for different traits) to examine recombination between markers for fine mapping.

### Gene Expression Analysis Using RT-PCR and qRT-PCR

Gene expression was studied by RT-PCR. Total RNA from independent tissues or embryos was isolated using TRIzol reagent (Invitrogen). Two micrograms of total RNA were treated with DNase I (Roche) and then used for cDNA synthesis using Reverse Transcriptase M-MLV (Promega). Expression of genes of interest in different tissues and in different developmental stages was firstly examined using RT-PCR with gene-specific primers. The relative expression of candidate genes was then studied using real-time RT-PCR (qRT-PCR) using KAPA SYBR FAST qPCR Kits (KapaBiosystems) with CFX96 Touch Real-Time PCR Detection System (Bio-Rad). Three replicates were performed for each sample and cDNA from 50 ng of total RNA was used for each reaction. *Beta actin* or *EF1A* was used as endogenous reference according to their expression stability. The 2^−ΔΔCT^ method (Livak and Schmittgen 2001) was used to quantify relative gene expression.

### Luciferase Reporter Assay

Candidate enhancer regions with closely flanking sequences from different alleles were cloned and constructed into the enhancer region of pGL3-Promoter vector that contains a basal SV40 promoter sequence (Promega). The reporter gene constructs together with pRL Renilla Luciferase Control Reporter Vector (Promega) were cotransfected into a Singapore grouper embryonic cell line (Chew-Lim et al. 1994) using TurboFect or Lipofectamine 3000 (Thermo Fisher). Luciferase activity was measured at 48 h post-transfection using Dual-Luciferase Reporter Assay System (Promega). Three independent transfections were carried out in six-well plates with each measured in triplicates.

### Enhancer Reporter Assay

Candidate enhancer sequences of different alleles with closely flanking sequences were constructed into ZED Vector (Bessa et al. 2009) using Gateway Recombination Cloning Technology (Thermo Fisher Scientific). T7-Transposase (Khattak et al. 2014) (Addgene) was transcribed using mMESSAGE mMACHINE T7 kit (Life Technologies), according to the manufacturer’s instructions. A final concentration of 40 ng/µl ZED constructs, 50 ng/µl transposase mRNA, and 0.05% phenol red were coinjected into one-cell stage embryos. The embryos were imaged for GFP and internal control red fluorescent protein (RFP) expression at different time points using a Leica MZFLIII microscope. The elements were considered as candidate enhancers if there were more than 20% of injected embryos showing consistent expression pattern of GFP at the presence of RFP (Bessa et al. 2009; Sharma et al. 2015).

### Knockout Using CRISPR/Cas9

CRISPR/Cas9 was used to introduce mutations into the genes or elements of interest. Guide RNA (gRNA) was designed using E-CRISP (Heigwer et al. 2014). gRNA sequences were blasted against the reference genome to avoid off-targets. Template of gRNA was assembled using PCR according to a previous method (Vejnar et al. 2016). In brief, gRNA was designed with common flanking adaptors as follows: 5′-TAATACGACTCACTATA[GGN(18)]GTTTTAGAGCTAGAA-3′. A universal primer was used to assemble gRNA temple with direct PCR, with the following sequences: 5′-AAAAGCACCGACTCGGTGCCACTTTTTCAAGTTGATAACGGACTAGCCTTATTTTAACTTGCTATTTCTAGCTCTAAAAC-3′. gRNA was transcribed using HiScribe T7 High Yield RNA Synthesis Kit (NEB) with 150 ng of purified DNA template and gRNA was subsequently purified using miRNeasy Mini Kit (Qiagen), according to the manufacturer’s instructions. Cas9 Nuclease NLS (NEB) and gRNA with a final concentration of 100 and 200 ng/µl, respectively, were coinjected into one-cell stage embryos. Both phenotypes and genotypes were screened for candidate mutants. DNA fragments spanning the targeted sequences of gRNAs were amplified using fragment-specific primers. PCR products were purified using QIAquick PCR Purification Kit (Qiagen) for mutant screening using T7 endonuclease assay (NEB). PCR products that showed cleavage in T7 endonuclease assay were then validated by TA cloning and Sanger sequencing. We developed a whole protocol for transgenic and CRISPR knockout technology for the fighting fish, a species of particular mating and brooding behaviors (supplementary note 3, Supplementary Material online).

### Ethics Declarations

All procedures for handling of fish were according to the instructions of the Institutional Animal Care and Use Committee (IACUC) of Temasek Life Sciences Laboratory, Singapore (Approval no. TLL (F)-16-003).

## Supplementary Material

Supplementary data are available at *Molecular Biology and Evolution* online.

## Data Availability

The genome sequences and related annotations of fighting fish are hosted by the web server of Temasek Lifesciences Laboratory (https://genhua.tll.org.sg/) and archived in China National GeneBank (CNGB) and DDBJ with BioProject accession nos. CNP0001745 and PRJDB7253, respectively. Sequences used for whole-genome sequencing, RNA sequencing, and RAD sequencing are available with the DDBJ Sequencing Read Archive (SRA) through BioProject ID PRJDB7253–PRJDB7255.

## Supplementary Material

msab110_Supplementary_Data
