# Supplementary material for: Genomic Basis of Striking Fin Shapes and Colors in the Fighting Fish
Source: Mol Biol Evol. 2021 Apr 19;38(8):3383–96. doi: 10.1093/molbev/msab110 (PMC8321530; doi:10.1093/molbev/msab110)
Supplement: msab110_Supplementary_Data [file msab110_supplementary_data.zip › Supplementary_information_MBE01_R1sub.pdf]

Supplementary information for the paper entitled

Genomic basis of striking fin shapes and colours in the fighting fish

Le Wang[1#], Fei Sun[1#], Zi Yi Wan[1$], Baoqing Ye[1$], Yanfei Wen[1], Huiming Liu[1], Zituo Yang[1], Hongyan Pang[1], Zining Meng[2], Bin Fan[3], Yuzer Alfiko[4], Yubang Shen[5], Bin Bai[1], May Shu Qing Lee[1], Francesc Piferrer[6, *], Manfred Schartl[7,8, *], Axel Meyer[9, *] & Gen Hua Yue[1,10,11 *]

[1] Molecular Population Genetics & Breeding Group, Temasek Life Sciences Laboratory, Singapore 117604, Singapore

[2] School of Life Sciences, Sun Yat-sen University, Guangzhou 510275, China

[3] Department of Food and Environmental Engineering, Yangjiang Polytechnic, Yangjiang 529500, China

[4] Biotech Lab, Wilmar International, Jakarta, Indonesia

[5] Key Laboratory of Exploration and Utilization of Aquatic Genetic Resources, Shanghai Ocean University, Shanghai 201306, China

[6] Institute of Marine Sciences (ICM), Spanish National Research Council (CSIC), 08003 Barcelona, Spain

[7] Developmental Biochemistry, Biocenter, University of Wuerzburg, 97074 Wuerzburg, Germany

[8] The Xiphophorus Genetic Stock Center, Department of Chemistry and Biochemistry, Texas State University, San Marcos, Texas 78666, USA

[9] Department of Biology, University of Konstanz, 78457 Konstanz, Germany

[10] Department of Biological Sciences, National University of Singapore, Singapore 117543, Singapore

[11] School of Biological Sciences, Nanyang Technological University, Singapore 637551, Singapore

[#] Contributed equally to this study

[$] Contributed equally to this study

[*] Corresponding authors

FP: piferrer@icm.csic.es

MS: phch1@biozentrum.uni-wuerzburg.de

AM: axel.meyer@uni-konstanz.de

GHY: genhua@tll.org.sg

Table of contents

1 Supplementary notes (pages 3 - 9)

Note 1: ***Estimation of genome size (page 3)***

Note 2: ***Measurement of Betta Coloration as a Quantitative Trait (page 4 - 5)***

Note 3: ***Breeding and microinjection of fighting fish (page 6 - 9)***

2 Supplementary figures for the main text (pages 10 - 29)

3 Supplementary tables for the main text (pages 30 - 43)

4 References (page 44)

1 Supplementary note

Note 1.

Estimation of genome size

We estimated the genome size of the fighting fish using three methods. The first was to use the k-mer-based calculation by ALLPATHS-LG (Gnerre, et al. 2011). The second was to apply the *de novo* assembled RADtags. RADtags were *de novo* assembled using *ustacks* (-m 3 -M 4) and *cstacks* (-n 4) in Stacks package (Catchen, et al. 2013). We then mapped the RADtags to scaffolds to estimate the proportion of mapped RADtags to all assembled RADtags (proportion (p) = 95.82%) and thus infer the missing sequences. The genome size can be estimated by S/p, where S is the size of scaffold assembly. The third one was to use a real-time PCR-based method (Wilhelm, et al. 2003) to quantify the genetic elements in a known amount of genomic DNA. High-quality genomic DNA was isolated using MagAttract HMW DNA Kit (Qiagen, Germany) and quantified using NanoDrop ND-3000 Fluorospectrophotometer (Thermo Fisher Scientific, USA), Qubit dsDNA HS Assay Kit (Thermo Fisher Scientific, USA) and Agilent Bioanalyzer 2100 (Agilent, USA). Only the DNA samples showing little variation in measurement among the three instruments (< 5%) were used for analysis. Three starting DNA samples (20 ng, 50 ng and 100 ng) were used for PCR to amplify a single copy locus in haploid genome. PCR products were purified and quantified, and then diluted to a series of DNA standard. A set of nested PCR primers were designed within the first round PCR product to quantify the initial target concentrations of both standard and unknown targets using real-time PCR with KAPA™ SYBR® FAST qPCR Kits (KapaBiosystems, USA) on CFX96 Touch™ Real-Time PCR Detection System (Bio-Rad, USA) according to manufacturer's instructions. C_T values are proportional to the logarithms of the initial target concentrations. The diluted standard was used to calibrate and estimate the concentrations of the unknown targets. Haploid genome size was estimated using the formula $C= m*N_A/(N*M_{BP})$, where m is the weight of starting DNA material, N is the number of copies of target in starting DNA material determined by real-time PCR, N_A is Avogadro's number, and M_{BP} is average molar mass of a base pair.

Table N1 Summary statistics of the genome size estimate based on ALLPATHS-LG, *de novo* RAD sequencing and real-time PCR. The genome size was estimated to be 436.9 ± 17.2 Mb based on the above three methods, suggesting that not more than 6.5 % of sequences were missing from the genome assembly with a size of 429.4 Mb for the yellow single tail female fish.

Method	Estimated genome size
ALLPATHS-LG (k-mer based)	436.8 Mb
De novo RADtags mapping	407.1 Mb
Real-time PCR	466.8 Mb
Mean ± SE	436.9 ± 17.2 MB

Note 2.

Measurement of Betta Coloration as a Quantitative Trait

We developed a protocol to quantify the pigments. However, due to the overall complexity of the main body, we could only accurately quantify red pigments in the caudal fin and head. To quantify the coloration of Betta, we measured the proportion of each fish's body that is covered by one of four types of colour cells according to Lucas (Lucas 1968): black, iridescent, red and yellow.

Firstly, each fish was placed on a black surface under a microscope (Leica MZFLIII), and detailed photographs of different body parts were taken with a Nikon digital camera DXM1200F. In this study, only the head and tail images were used in the final colour quantification, as the distribution of pigments in these areas was much clearer and it was harder to perform the appropriate segmentation on the images of the other parts due to higher complexity. Segmentation was performed on each image using the Python version of the image processing package OpenCV (available at: https://pypi.org/project/opencv-python/).

The following are the main steps we carried out:

1. First, Otsu Binarization was applied on the smoothness, saturation and brightness matrices of the image, then the results were combined to separate the fish body from the background. Thereafter the background was excluded from further analysis.
2. Then, we applied some basic image morphological functions to fill up holes in the fish body part, or remove tiny persistent features in the background that could not be removed by the previous step.
3. Within the region of interest, we identified the pixels that corresponded to black, iridescent, red or yellow cells. The remaining pixels were also categorised into: pale (i.e. area with no pigment), specular (i.e. white high light due to reflection of light) and pitch black (e.g. pupil of eye). All pixels within the same category were replaced with single colour (e.g. all "red cell" pixels replaced with bright red).
4. After all the above segmentations, we took the resulting image and counted the number of pixels of each colour in the fish body part.
5. Finally, for each colour (x) of interest, its quantification was done as follows:
   Proportion of x = number of x pixels / (total number of body pixels)

Our method of colour quantification was based on a few observations we made about the images:

a) In each image, the background tended to be smoother, darker and have higher saturation than the fish body part.
b) Each image was detailed enough that we could see discrete pigments of colour. We could use these features as reference to adjust our detection parameters for detecting each type of colour cell.

The above steps were implemented in the Python code contained in **Supplementary script (for_phenotyping_red_pigments).py**, which has been attached as the supplementary files**.**

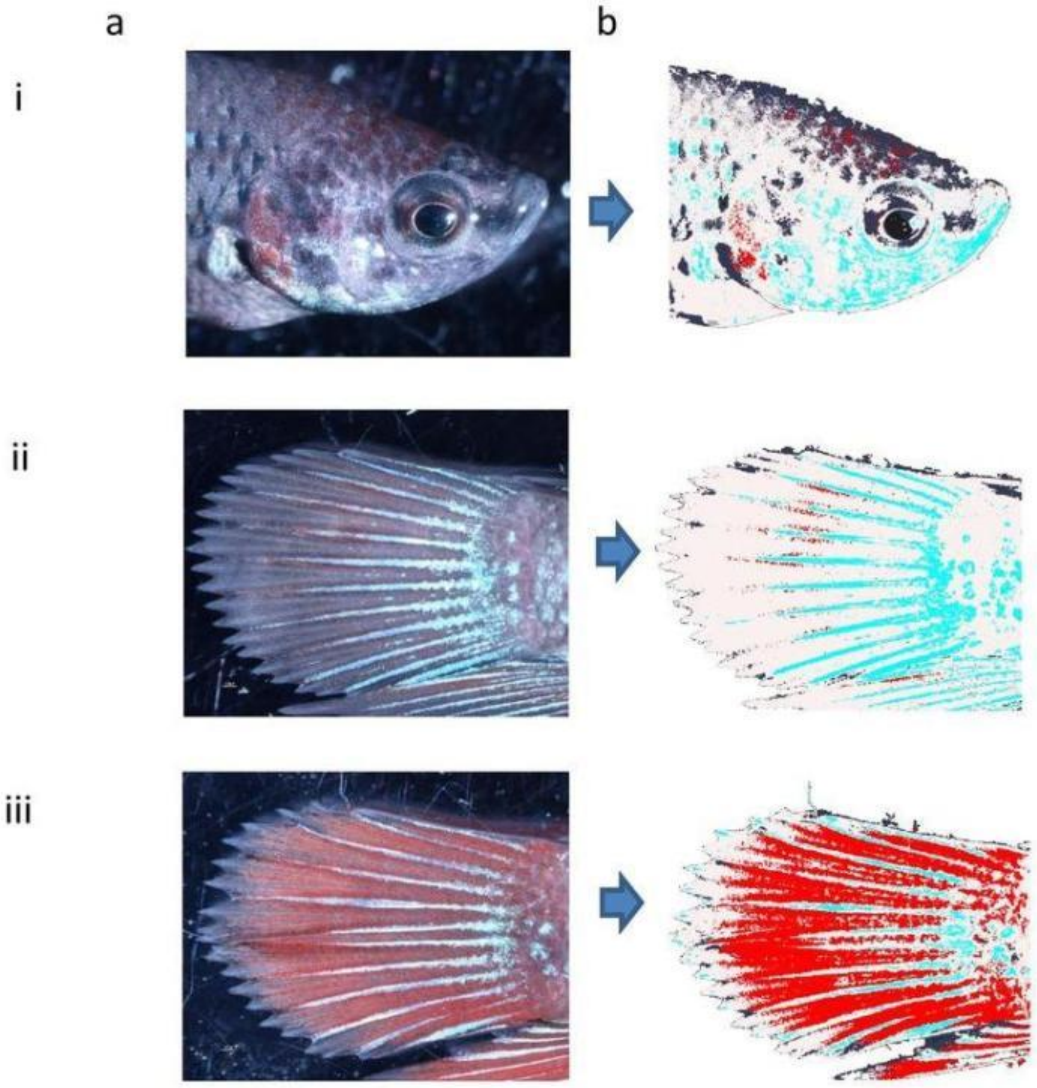

c

Feature in a	specular	iridescent	red	black	yellow	pale	Pitchblack	background
Colour in b								(transparent)
Proportion in b:								
i	0.00714	0.08033	0.01737	0.21214	0.00004	0.67305	0.009928	(excluded)
ii	0.00441	0.15450	0.01157	0.02422	0.00013	0.80514	0.000021	
iii	0.01328	0.06034	0.45664	0.04118	0.00029	0.42794	0.000325	

Figure N1 Image of head and tails of fish, before (**a**) and after segmentation (**b**). (**c**) shows the colour coding for each category of pixels, and its quantification for different colour categories in individual samples.

Note 3.

Breeding and microinjection of fighting fish

Due to the unique courtship, mating, and bubble-nesting behaviour of fighting fish, the standard procedures of CRISPR genome modifications developed in other fish had to be adjusted. Because of the extensive spawning behavior, almost all the collected eggs were already at late one-cell stage, which might have reduced the efficiency of genetic modifications. Another challenge is that development of embryos of fighting fish needs special parental care in bubble nest and "mouthing" behavior of the care-giving father. Development in artificial conditions significantly reduces the survival rate of embryos. We observed that the one-month survival rate after microinjection was no more than 5 %, limiting observation of phenotypes. Here, we introduced some strategies to improve the efficiency of CRISPR in this fish of special mating behaviors, as below.

Description of culture, breeding, egg collection and microinjection of fighting fish

To know the Betta

Before breeding any species, it is important to know as much information as possible about the species. Betta is a large genus of small, often colourful, freshwater ray-finned fishes in the gourami family. Its origins can be traced back to the 1800s in Southeast Asia, including Malaysia, Thailand, Cambodia and Vietnam, etc. There are currently 73 recognized species in this genus. The well-known species is *B. splendens*, commonly known as the Siamese fighting fish. We just call it Betta in this instruction.

In the same species, male Bettas mostly have larger and longer bodies, more bright colours and longer fins, and are much more aggressive than females. There are also other sexual differences, including egg spot, horizontal fear stripes and vertical breeding bars in the female, and beard, bubble nest making and babysitting behaviour of the male, etc. Betta has many different colour variations due to different layers of pigmentation in their skin. The layers consist of yellow, red, black, iridescent (blue and green), and metallic. Any combination of these layers can be present, leading to a wide variety of colours (Simpson 1968). Betta belongs to the suborder Anabantoidei which has a defining characteristic: the labyrinth organ, a many-folded accessory breathing organ. This organ allows Betta to take in oxygen directly from the air and thus survive in oxygen depleted waters. Labyrinth fish are also well known for their bubble-nesting behaviour, although not for all species. Some other species in the genus show mouth nesting behaviour in contrast to bubble nesting of *B. splendens* (Rüber, et al. 2004). All these features very likely have significant influence on sexual selection in this species and play critical role in artificial breeding of Betta. According to our experience, the mating success rate for a randomly selected pair of parents is very low. Selection based on prior tests is necessary.

Culturing the Betta

We used zebrafish culture system for our permanent Betta tanks. All the Bettas imported from outside of lab were firstly kept in 1 separate system for selection before use, in case there is pathogen infection. Another 3 systems were used for our self-bred Betta families or individuals. These systems were mainly used to keep selected parents for breeding, fry after 1month old, useful mature males separated to prevent fighting, or sampled individuals for experimental use, etc. We also had 15 50-L square PVC tanks with trickle filter system as our permanent Betta tanks. 13 of them were used for Betta fry culturing or rearing the families. Another two were used to pre-treat and store fresh water to supply to breeding tanks and tanks with fry. The zebrafish housing systems with lids, with each tank of 10L, were used as the betta breeding tanks in our project. The tanks were filled with 2/3 fresh water and directly put on the table without water flow. A floating transparent plastic jar lid with dimeter of 10 cm was placed on the water surface to simulate the habitats for construction of bubble nests for the males.

Choose the breeding parents

According to different experiment designs, it was necessary to choose the breeding pairs with specific traits to breed. For example, when we set up crosses for double-tail mutant study, we need to cross a homozygous single-tail Betta with a homozygous double-tail Betta to get the F1 family with all heterozygous single-tail individuals. Then a pair of F1 individuals was selected to cross for F2 family with all 3 genotypes.

In addition, there are also some basic rules to follow to choose the breeding pair. The selected breeders should be mature with ideal age of 4-12 month old. They should be actively swimming with energy and vigour in the original tank with no sign of disease, infection or fin damage. They should be roughly the same size, and the female should be slightly smaller than the male. The male should have bright coloration and long and undamaged fins. If the selected males can build bubble nests in the original tanks, these fish would be selected as candidates. It is suggested that this type of male is ready to mate. Females should have a visible white spot behind the ventral fin, called "egg spot". Depending on coloration type, females may also display vertical stripes on her body when it is ready for breeding.

Setting cross

The selected male will be added to the breeding tank first. Then the female will be introduced to him within a transparent beaker at the center of the tank. The water line of the tank should be lower than the top of the beaker. Then we observe their behaviour to see if they are interested in each another. Usually the male will swim, displaying his fins, flaring his gills and twisting his body in a dance to show off, and then start to build a bubble nest just around or under the transparent jar lid. The female will darken in colour (if any), display vertical stripes on her body and angle her head down submissively. Or else, we would have to change the combination between male and female for further examination of sexual selection.

Once the bubble nest is built, the female will be released from the beaker into the tank. Normally the male will start chasing the female around the tank and try to engage her by showing off or performing a mating "dance". Sometimes it will become a fight or bullying. If

the male (or sometimes the female) is too aggressive and bad physical damage occurs, we will stop the breeding process for the pair and continue testing the other combinations.

If they show interest in each other, the female will swim under the bubble nest and then they will embrace, with male wrapping his body around the female. It may take a few times until the first egg release. During the embrace, both fish will be temporarily motionless as the female releases eggs and the male releases sperm to fertilize them. The number of eggs in each release is different, ranging from 1 to 40, as per our observation. During the embrace, the female will be in a brief motionless state while the produced eggs will fall to the bottom of the tank. Then the male will swim down to retrieve the sinking eggs and deposit them into the bubble nest with his mouth. Sometimes the female will also help with this after recovering, but sometimes they also possibly eat the eggs. They will repeat this act until the female stops releasing eggs. Once this process is disturbed, the mating would be stopped and the eggs could be eaten out. Hence it is particularly important to avoid disturbing them, as far as possible, when collecting eggs for microinjection.

The whole breeding will last from minutes to hours, typically 2-3 hours. Total produced egg number varies from dozens to 700+ according to our records. When finished, the male will chase away the female from his territory and start to take care of the eggs by repairing the bubble nest which was possibly damaged during the mating, or picking up the falling eggs and sticking them back to the nest. According to our experience, such paternal care can significantly increase the offspring survival rate during embryo development.

Microinjection

If the cross is planned to produce eggs for microinjection, we will use a transparent Pasteur pipet to carefully collect the eggs into a small beaker, without interrupting the breeding progress. Because the parents would display dancing style behaviour and special parental care of eggs as described above before each spawning, we have to wait until the parents pick up the eggs and put them into the bubble nest. This process can significantly influence the fertilization rate. Due to these reasons, the eggs will be collected every 15mins to make sure they are still within single cell stage before first cleavage. The eggs are then aligned into grooves in agarose gel contained in a petri dish, which gently hold the eggs in place to facilitate microinjection. After microinjection, the eggs will be gently washed out from the grooves of the agarose gel into a beaker with shallow (< 2cm) egg-water. In natural environment, the eggs would be in the bubble nest in the water surface. Similarly, the shallow water can guarantee the oxygen concentration is sufficient for embryo development. The beaker containing eggs will be kept in the temperature of 28°C for development.

Egg hatching, embryo culture and feeding

In normal mating experiments not destined for microinjections, after the mating, we would remove the female from the tank and keep the male there to take care of the eggs until hatching. The newly hatched embryos will remain in the bubble nest until the full absorbing of their yolk sacs in 2-3 days post hatch. If left in the tank, the male will continue taking care of the eggs or fry until the latter are free swimming. Then the male will be removed from the breeding tank.

During this period, no feeding and disturbing of the male baby-sitter is allowed, or it will start to eat the eggs/fry.

For microinjected eggs, after the procedure described in the previous section, the embryos would be kept in the beaker at 28°C for development until feeding. However, due to lacking of parental care, survival rate for the microinjected embryos is rather low compared to the controls with the male baby-sitter.

As soon as the fry are free swimming, newly-hatched decapsulated brine shrimp are fed to them until one month old. Then 0.3 mm pelleted food will be introduced to them gradually, reducing the amount of brine shrimp until only pelleted food is given. When they are big enough, blood worm and 0.5 mm pelleted food will be feed to them twice a day until maturity.

2 Supplementary figures for the main text

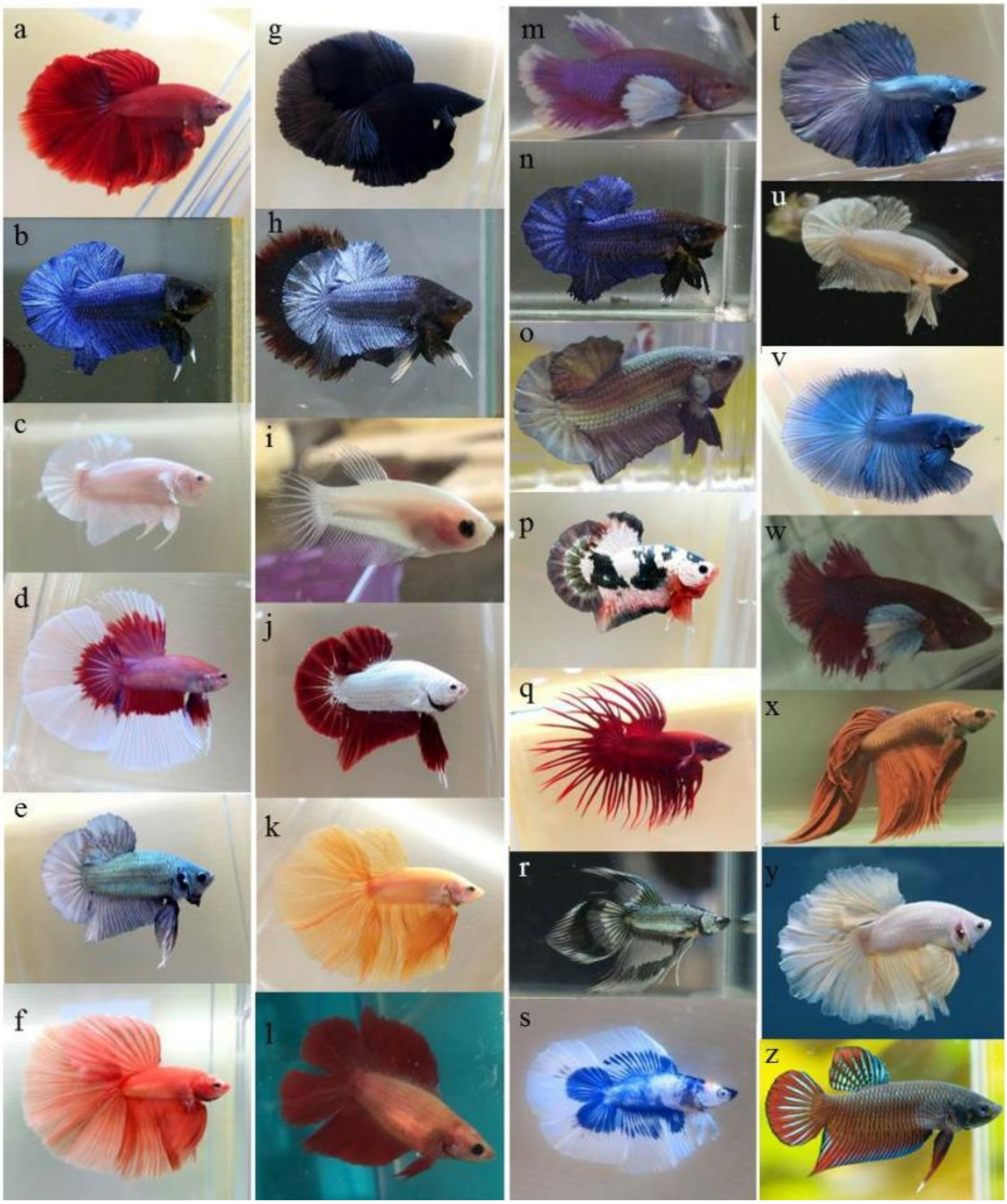

Figure S1. Representative varieties of diverse body morphology in the fighting fish, including pigmentation varieties (**a**-**p**) and finnage varieties (**q**-**y**): (**a**) red; (**b**) royal blue; (**c**) white; (**d**) butterfly; (**e**) the Turquoise; (**f**) orange; (**g**) black; (**h**) steel blue; (**i**) transparent; (**j**) the Dragon; (**k**) yellow; (**l**) the Cambodian; (**m**) purple; (**n**) spotted fin; (**o**) copper; (**p**) the Koi; (**q**) crown tail; (**r**) spade tail; (**s**) double tail; (**t**) super delta; (**u**) the Plakat; (**v**) the half-moon (**w**) big ear; (**x**) the Veiltail and (**y**) rose tail, in comparison to the wild type (**z**) (Monvises, et al. 2009). Photos of the varieties are provided by the Betta Club Singapore and the photo of the wild type is from Monvises et al. 2009 (Monvises, et al. 2009).

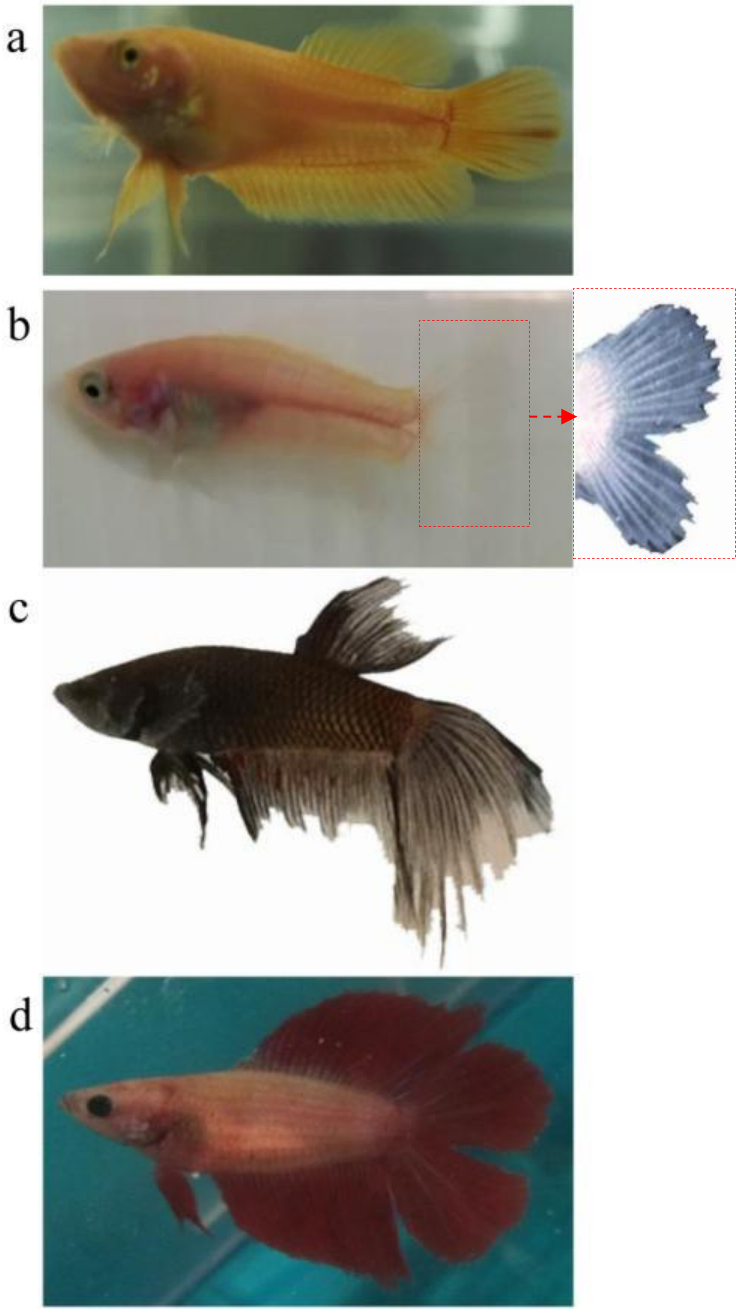

Figure S2. Fighting fish individuals used for whole-genome sequencing. (a) a yellow homozygous single-tail and albino female; (b) a transparent double-tail and albino male, along with clearer image of its tail (c) F0B1male, a black homozygous single-tail and homozygous melanin male and (d) DtY2female, a Cambodian double-tail and albino female. The genotypes of each fish were determined according to both the cross tests and the phenotype-associated DNA markers obtained in the following mapping and association tests.

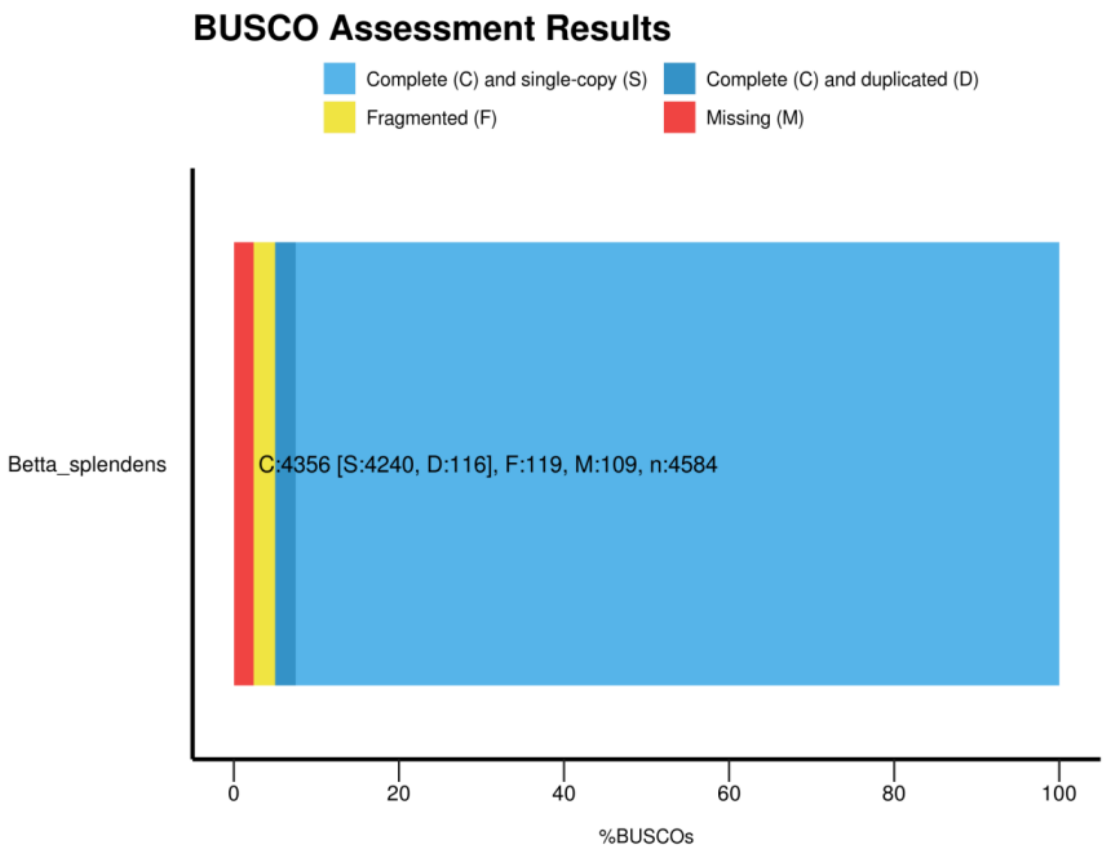

Figure S3. Genome assembly completeness (female *de novo* assembly) as evaluated by BUSCO. In summary, 119 (2.6%) and 109 (2.4%) genes were estimated as fragmented and missing, respectively, while the remaining 4356 out of 4584 (95.0%) tested genes were complete.

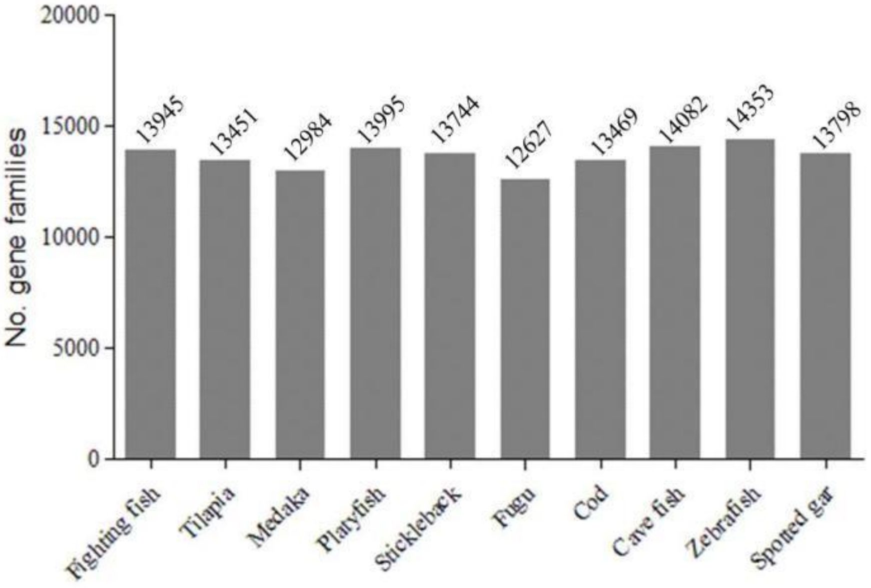

Figure S4. Comparison of the number of gene families between the fighting fish and the other representative fish. These fish include Nile tilapia (*Oreochromis niloticus*), medaka (*Oryzias latipes*), platyfish (*Xiphophorus maculatus*), stickleback (*Gasterosteus aculeatus*), fugu (*Takifugu rubripes*), cod (*Gadus morhua*), blind cave fish (*Astyanax mexicanus*), zebrafish (*Danio rerio*) and spotted gar (*Lepisosteus oculatus*). The gene families were identified based on the Pfam protein families database (Bateman, et al. 2004). A total of 22,977 protein-coding genes were predicted for the fighting fish, which were classified into 13,945 gene families.

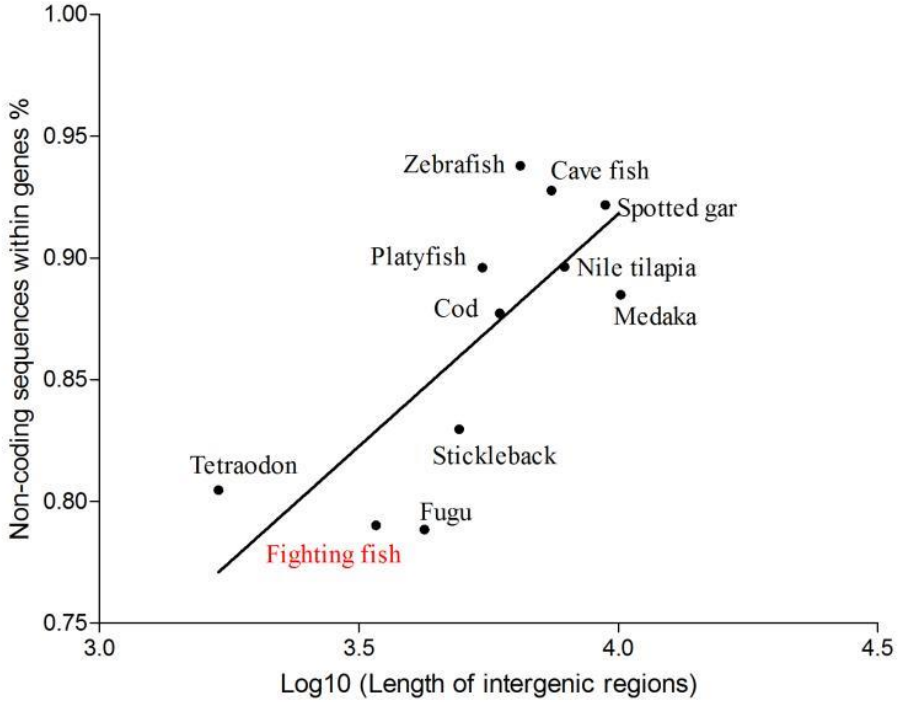

Figure S5. The overall length of intergenic regions and proportion of non-coding sequences within genes. The parameters were estimated based on genome assemblies and the corresponding annotations (Ensembl database release 86) for each representative fish species. The overall length of intergenic regions shows a significant linear correlation with the overall proportion of non-coding sequences in these fish, as measured using Pearson's correlation test (R = 0.76, $P < 0.01$). Linear regression model was used to calculate R squared value and goodness of fit ($R^2 = 0.58$, $P < 0.01$).

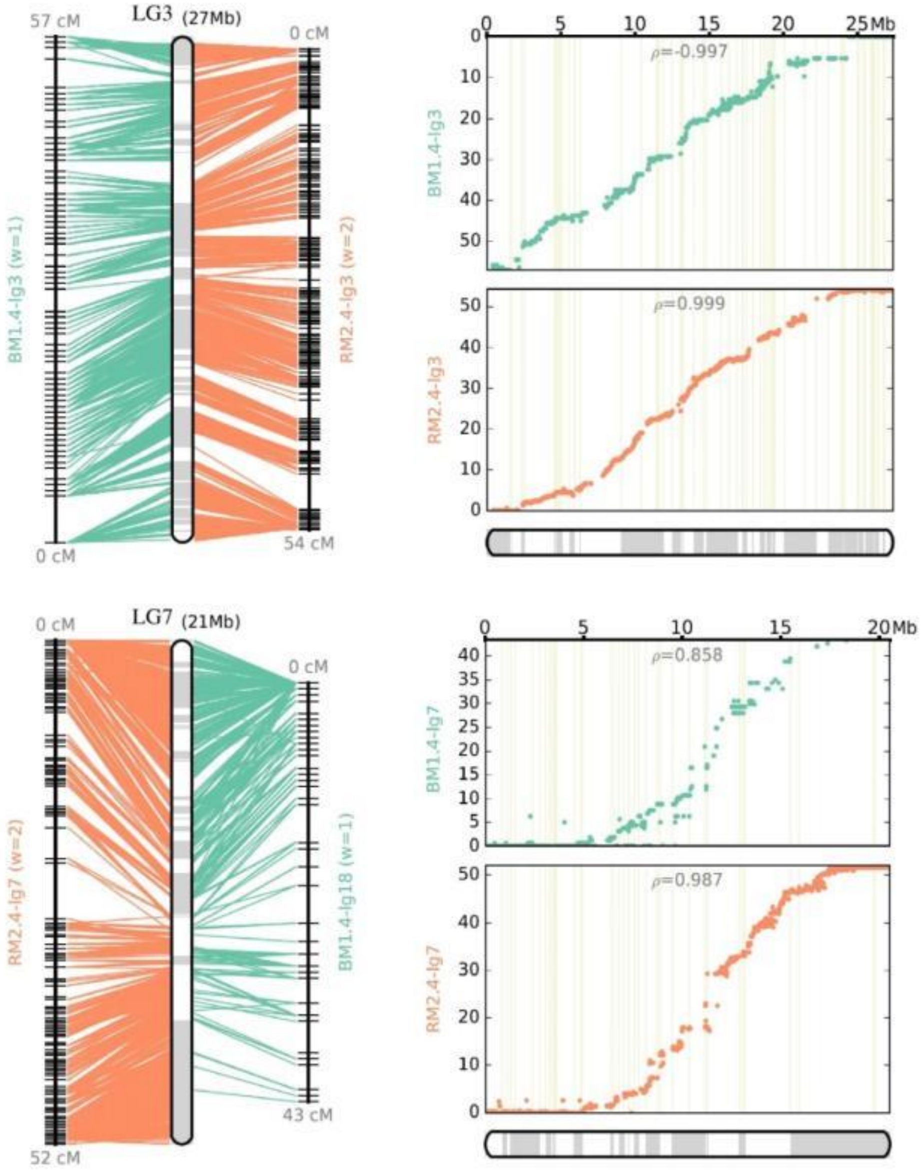

Figure S6. Anchoring scaffolds onto genetic maps of BM1 and RM2. The analysis were carried out using the program ALLMAPS (Tang, et al. 2015) with default parameters, where the RM2 sex-averaged map is given a higher weight than the BM1 sex-averaged map. Pearson's correlation coefficient between each linkage group and the corresponding physical maps is denoted with ρ value. Linkage groups showing the highest and the lowest overall ρ values are presented: LG3 and LG7, respectively.

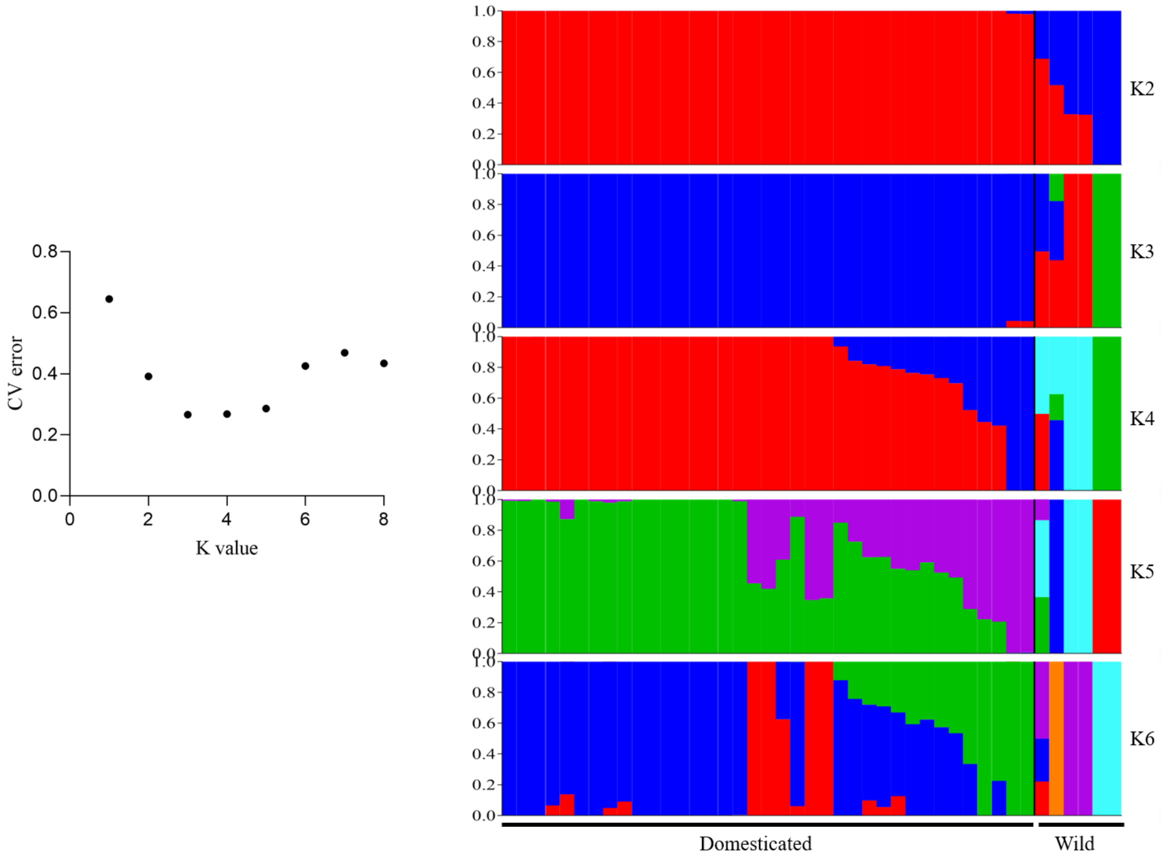

Figure S7. Inferring the most likely number of genetic clusters (K) and genetic structure at different K values, among wild and domesticated fighting fish.

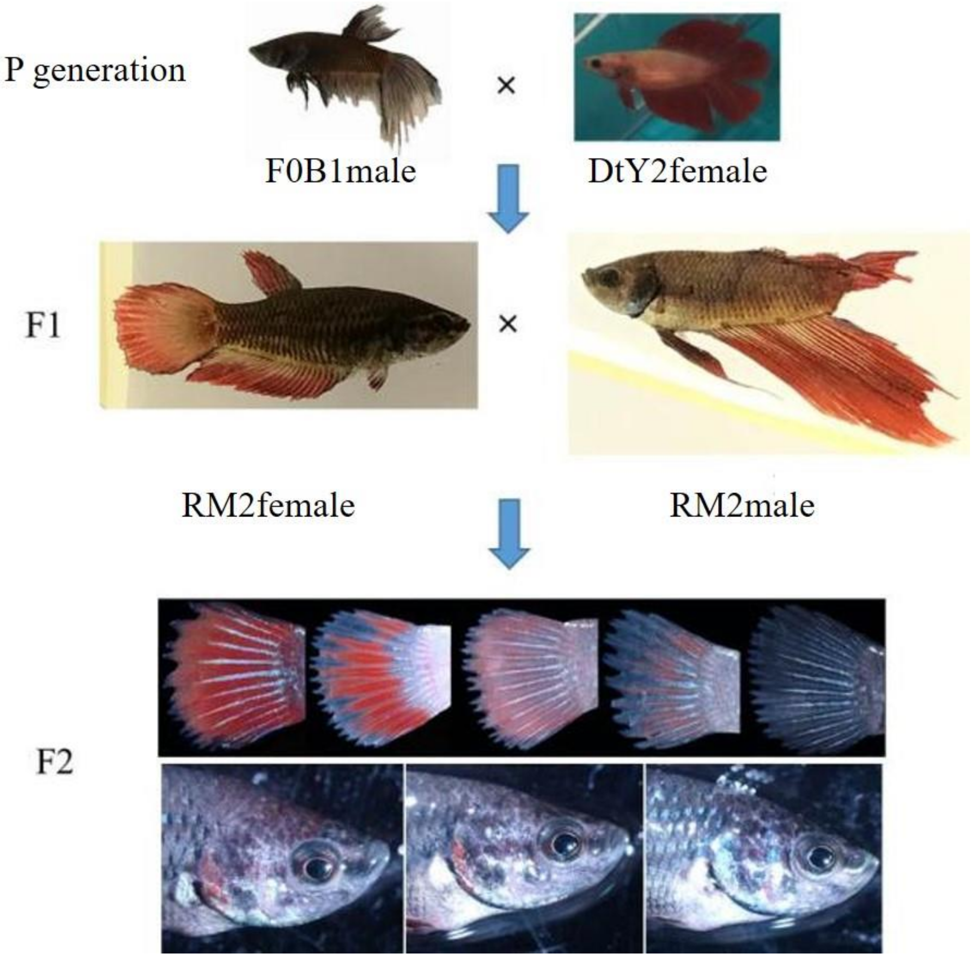

Figure S8. Phenotypic segregation of red pigmentation in the pedigree of F2 family RM2. In F2 individuals, remarkable variations in the distribution of red pigments in different body sections, such as tail and head, are observed.

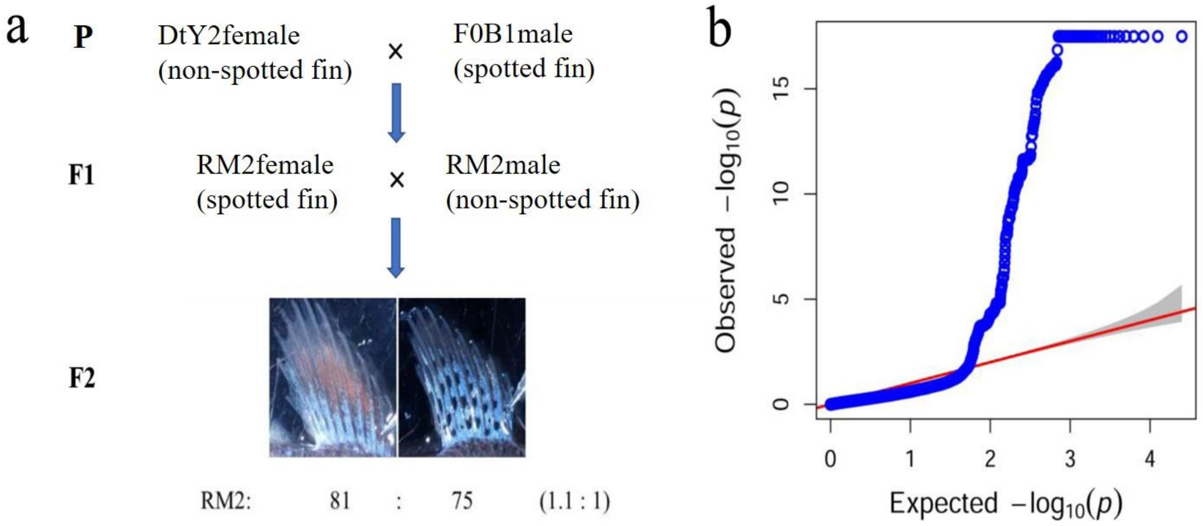

c

Phenotypes	Samples	12206865	12209486	12230137	12235095	12235216	12254488	12306974	12353135	12408542	12467998	12527033	12628449	12676772	12739143	12782216	12865015	12936047	13039206	13040187	13047258	13160178	13208197	13219529	13263414	13278572	13279888	13302125
Spot (Wildtype)	s239	1	1	1	1	0	0	0	0	0	0	0	0	0	0	0	0	0	0	0	0	1	1	1	1	1	1	1
Spot (Wildtype)	s266	1	1	1	0	0	0	0	0	0	0	0	0	0	0	0	0	0	0	0	0	1	1	1	1	1	1	1
Non spot	s090	0	0	1	1	1	1	1	1	1	1	1	1	1	1	1	1	1	1	1	1	1	1	1	0	0	0	0
Non spot	s165	0	0	0	1	1	1	1	1	1	1	1	1	1	1	1	1	1	1	1	0	0	0	0	0	0	0	0
Non spot	s200	0	0	0	1	1	1	1	1	1	1	1	1	1	1	1	1	1	1	1	1	1	1	0	0	0	0	0
Non spot	s205	1	1	1	1	1	1	1	1	1	1	1	1	1	1	1	1	1	1	1	1	1	1	0	0	0	0	0

Figure S9. Phenotypic segregation for spotted vs non-spotted dorsal fin in F2 mapping family RM2 of fighting fish. Due to the influence of iridescent pigmentation patterns and the albino locus, which also segregated in this mapping cross, only 156 individuals could be phenotyped. (**a**) Phenotypic segregation of spotted vs non-spotted dorsal fin in F2 family, RM2. (**b**) Q-Q plot for *P* values of genome-wide association study under compressed mixed linear model. (**c**) Recombinants around the genetic locus for spotted vs non-spotted dorsal fin, revealed by RAD sequencing-based SNP markers.

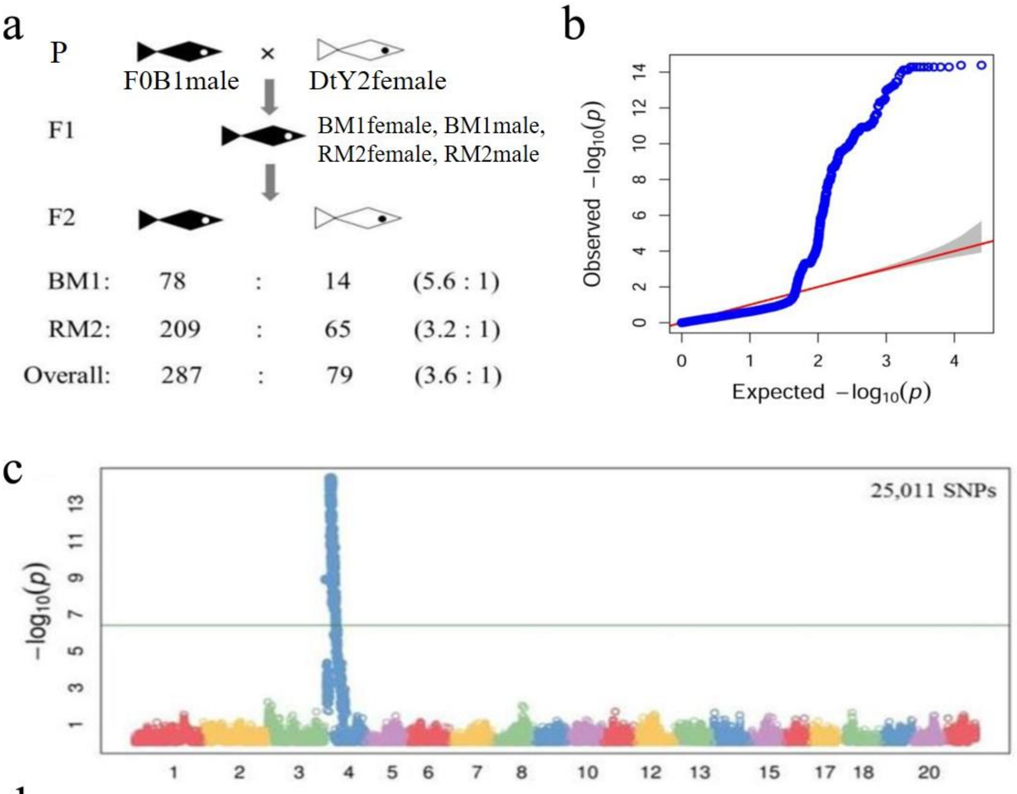

d

Phenotype	Sample	2424459	2478578	2478699	2505313	2640771	2816273	2830366	2858769	2874013	2893109	2914600	2930883	2959176	3001174	3005782	3039491	3048308	3053746	3060647	3064618	3078758	3079040	3090550	3144153	3151948	3155279	3162210	3193578	3194555
Albino	S051	1	1	1	1	2	2	2	2	2	2	2	2	2	2	2	2	2	2	2	2	2	2	2	2	2	2	2	2	2
Albino	S068	1	1	1	1	2	2	2	2	2	2	2	2	2	2	2	2	2	2	2	2	2	2	2	2	1	1	1	1	1
Albino	S069	0	0	0	2	2	2	2	2	2	2	2	2	2	2	2	2	2	2	2	2	2	1	1	1	1	1	1	1	1
Albino	S114	1	1	1	1	1	2	2	2	2	2	2	2	2	2	2	2	2	2	2	2	2	2	2	2	2	2	2	2	2
Albino	S146	1	1	1	2	2	2	2	2	2	2	2	2	2	2	2	2	2	2	2	2	2	2	2	2	2	2	2	2	2
Wildtype	S154	2	2	2	1	1	1	1	1	1	1	1	1	1	1	1	1	1	1	1	1	1	1	1	1	1	1	1	1	1

Figure S10. Phenotypic segregation and genome-wide association mapping for melanin vs albino in fighting fish. (**a**) Phenotypic segregation of wild-type pigmented and albino fighting fish in two F2 families: BM1 and RM2. The pedigree was set up by crossing homozygous wild-type pigmented and albino P generation fish. All F1 individuals showed melanin pigmentation while F2 fish show an overall melanin to albino ratio of 3.6: 1. The actual ratio of phenotypic segregation deviated slightly from the expected ratio of 3: 1. This may be explained by our observation that the albino fish are weak during development and that their mortality is significantly higher than wild type. (**b**) Q-Q plot for *P* values of genome-wide association study under compressed mixed linear model. (**c**) Genome-wide association mapping of the genomic locus for albinism using BM1 and RM2 families with the R package GAPIT (Lipka, et al. 2012). Cut-off value of genome-wide significance is denoted with a green line. Only one significant locus was identified at LG4. (Note, because of the influence of iridescences and red pigments, it is difficult to capture the melanin phenotype in whole-body photography. We then use the cartoon instead). (**d**) Recombinants around the genetic locus for albino mutant, revealed by RAD sequencing-based SNP markers.

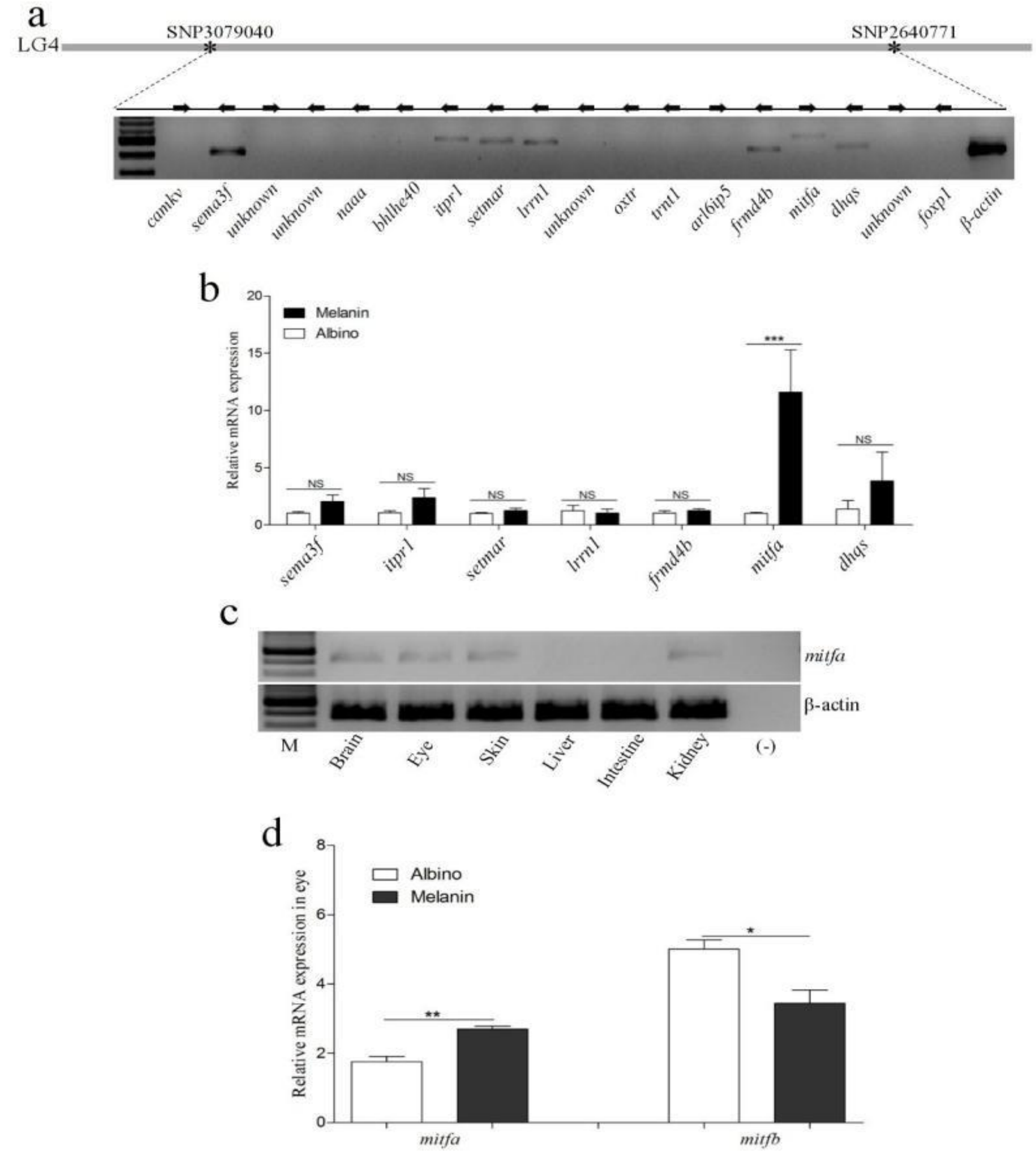

Figure S11. Fine scale mapping of the genomic locus for melanin vs albino and the expression patterns of predicted genes in the locus. (**a**) Genomic region/haplotypes showing no recombination between the two flanking SNPs within 293 genotyped individuals, genomic organization of 17 predicted genes within this region and the expression patterns of these 17 predicted genes in skin of melanin fish using reverse transcription PCR (RT-PCR) with gene specific primers. (**b**) Expression patterns of seven candidate genes showing expression in (**a**) between melanin and albino fighting fish. Differences are examined using Mann-Whitney tests (n=3 and *** denotes $P < 0.001$). (**c**) Expression pattern of the candidate gene, *mitfa,* in different tissues of fighting fish with melanin in skin as examined using RT- PCR with gene specific primers. House-keeping gene, *β-actin* was used as positive control. (d) Expression pattern of the paralogous genes, *mitfa* and *mitfb* in eye as examined using RT-PCR with gene specific primers. Differences are examined using Mann-Whitney tests (n=3 and ** and * denotes $P < 0.01$ and $P < 0.05$, respectively).

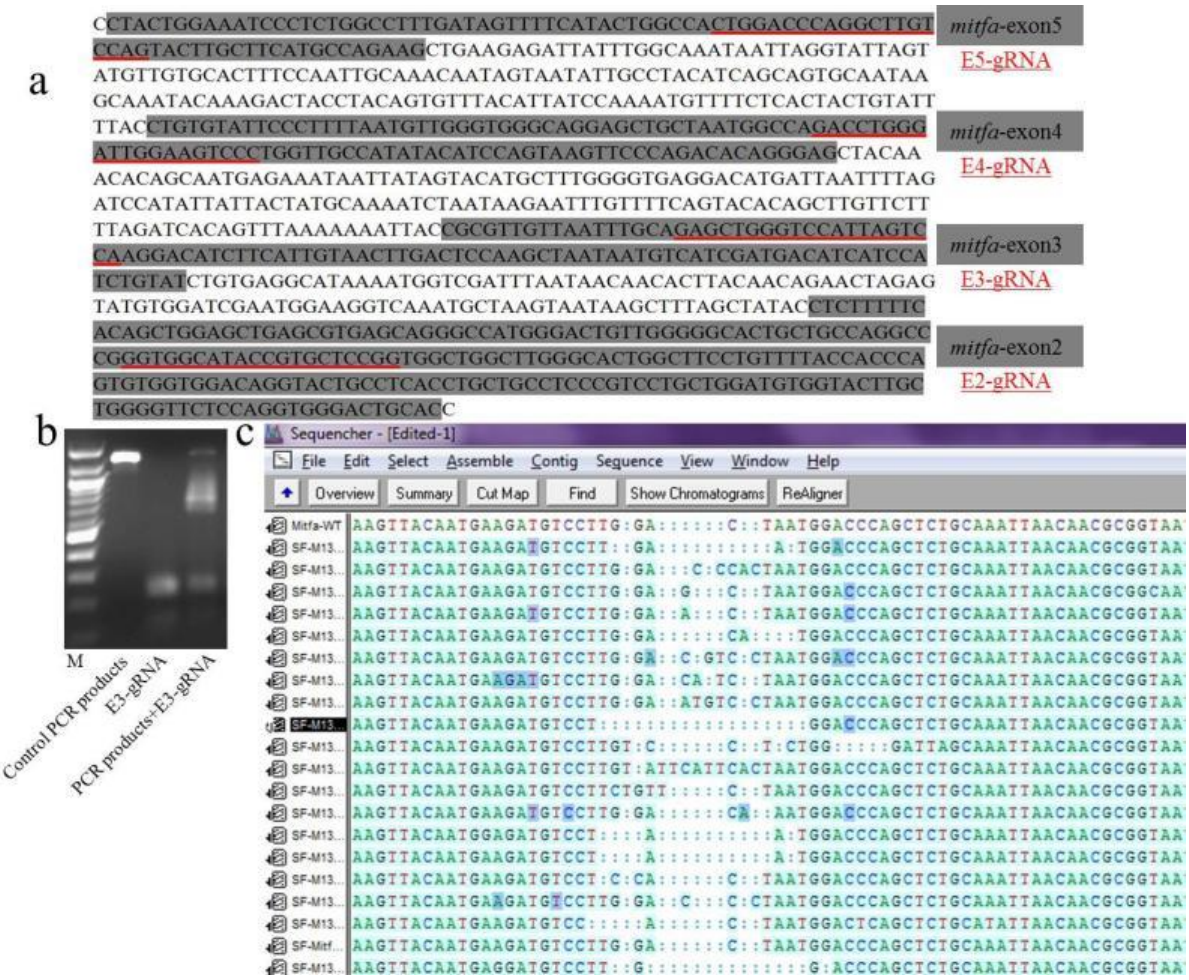

Figure S12. Design of gRNAs and CRISPR/Cas9 knockout of the *mitfa* gene in fighting fish. (**a**) Sequences of four gRNAs with targets in exon2, exon3, exon4 and exon5 (underlined) of *mitfa* gene. The exons are highlighted with dark gray shading. (**b**) In vitro test of the gRNAs using Cas9 Nuclease protein (NEB, #M0386M). PCR products of 1325 bp containing exon3 of *mitfa* of fighting fish are significantly digested by exon3-gRNA (E3-gRNA), while exon2-gRNA, exon4-gRNA and exon5-gRNA showed little evidence of digestion (data not shown). (**c**) Alignment of wild-type and mutant *mitfa* genomic sequences containing the E3-gRNA target sequences, generated by CRISPR/Cas9 system. Overall, ~ 90% of modified sequences are frameshift mutations. We screened 46 modified fish by T7 endonuclease assay after 48 hpf, when observation of phenotypes became feasible.

We sequenced the clones of two fish that showed no melanin-containing cells in skin and found more than 24 mutant alleles but no wild-type alleles in selected 96 clones. However, for the fish showing reduced melanized cell numbers in comparison to wild-type pigmented fish, a significant ratio of wild-type alleles was observed.

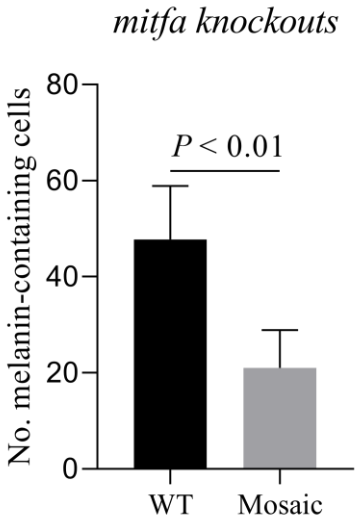

Figure S13. Estimated number of melanin-containing cells in the skin between wild-type controls and mosaic *mitfa*-knockout fish in fighting fish at 48 hpf. (n=4 and differences are examined using two-tailed t-test)

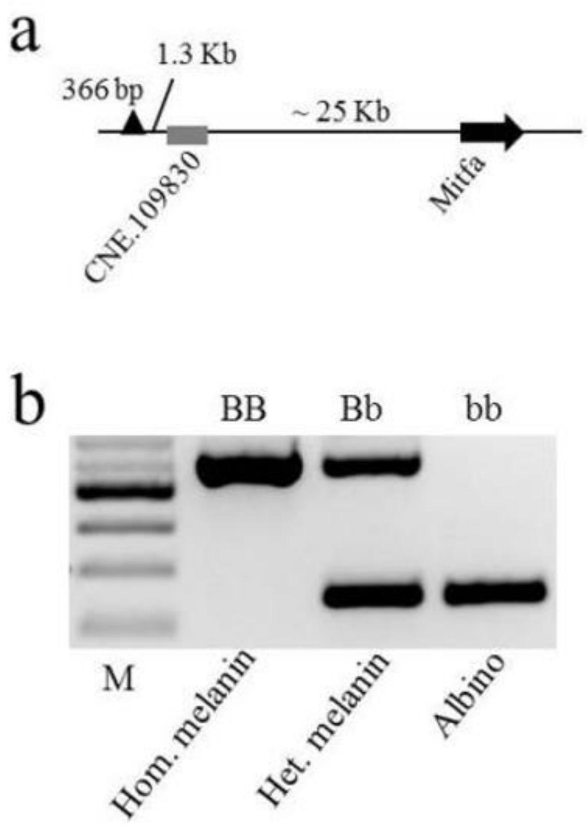

Figure S14. The deletion and its flanking CNE upstream of the *mitfa* gene. (**a**) Genomic organization of the 366-bp deletion at ~25-kb upstream of *mitfa* gene, where a conserved non-coding element (CNE.109830) is predicted at 1.3-kb downstream of the deletion. (**b**) Genotypes of the deletion are completely associated with phenotypes in 982 examined fish including 759 wild-type pigmented and 223 albino fish.

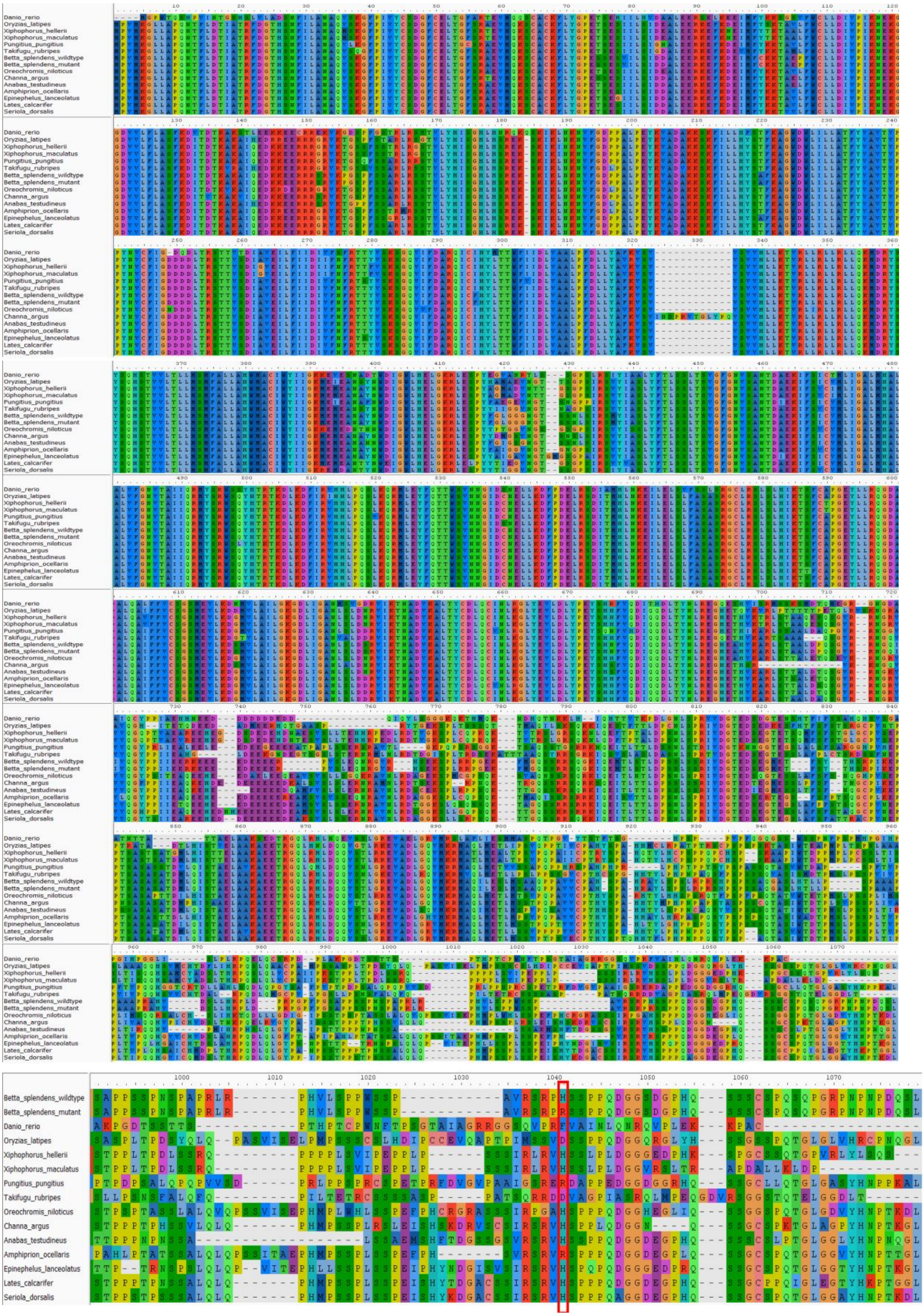
Danio_rerio
Oryzias_latipes
Xiphophorus_hellerii
Xiphophorus_maculatus
Pungitius_pungitius
Takifugu_rubripes
Betta_splendens_wildtype
Betta_splendens_mutant
Oreochromis_niloticus
Channa_argus
Anabas_testudineus
Amphiprion_ocellaris
Epinephelus_lanceolatus
Lates_calcarifer
Seriola_dorsalis

Figure S15. Alignment of protein sequences of Kcnh8 across 14 teleosts: *Betta splendens* (BSCG00000003509), *Xiphophorus hellerii* (XP_03243774), *Takifugu rubripes* (XP_003969315), *Oreochromis niloticus* (XP_003448945), *Lates calcarifer* (XP_018522184), *Oryzias latipes* (XP_011479155), *Pungitius pungitius* (XP_037330289), *Amphiprion ocellaris* (XP_023142853), *Epinephelus lanceolatus* (XP_033492221), *Anabas testudineus* (XP_026203881), *Seriola dorsalis* (XP_023282428), *Channa argus* (KAF3707579), *Lepisosteus oculatus* (XP_015212837) and *Danio rerio* (XP_017207899). Wild-type and mutant Kcnh8 sequences of *Betta splendens* are put at the top positions. Amino acid change in *Betta splendens* and its corresponding amino acids across studied teleosts, are highlighted in red box.

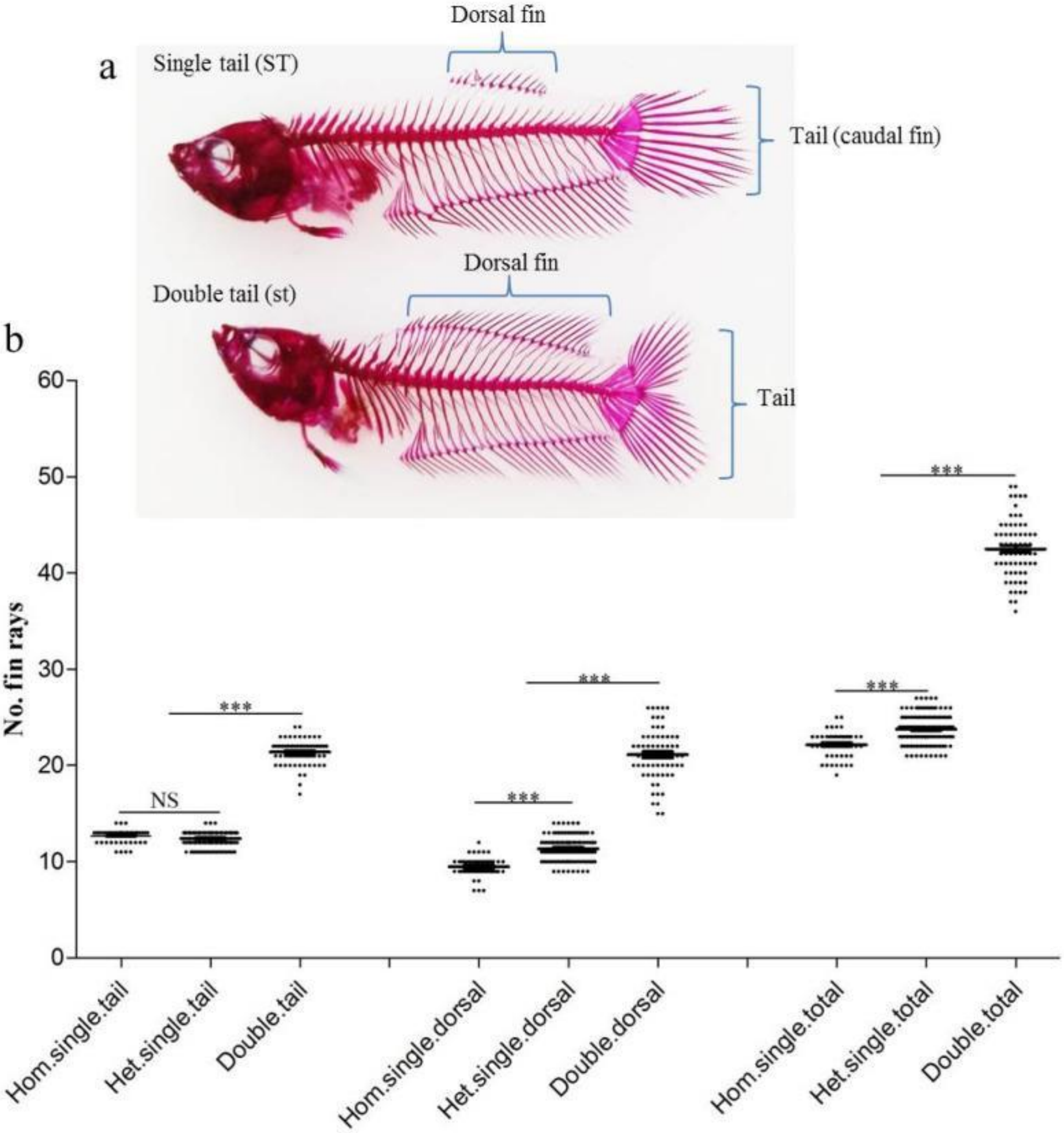

Figure S16. Characterization of finnage variations between single-tail (wild-type) and double-tail fighting fish. (**a**) Overview of dorsal fin and caudal fin variations between single-tail (wild-type) and double-tail fighting fish. The skeleton system was stained using Alcian blue and Alizarin red S according to a previous method (McLeod 1980). (**b**) Differences of the number of fin rays in dorsal fin and caudal fin among homozygous single-tail, heterozygous single-tail and double-tail fighting fish. The numbers of fin rays are significantly different between any pair of these three types of fish except for the caudal fin, where the numbers of fin rays between homozygous single-tail and heterozygous single-tail fish are not statistically different (Mann-Whitney: *, $P < 0.05$; **, $P < 0.01$; ***, $P < 0.001$). The genotypes are determined by both SNP markers identified in association mapping studies and the indel marker developed in this study.

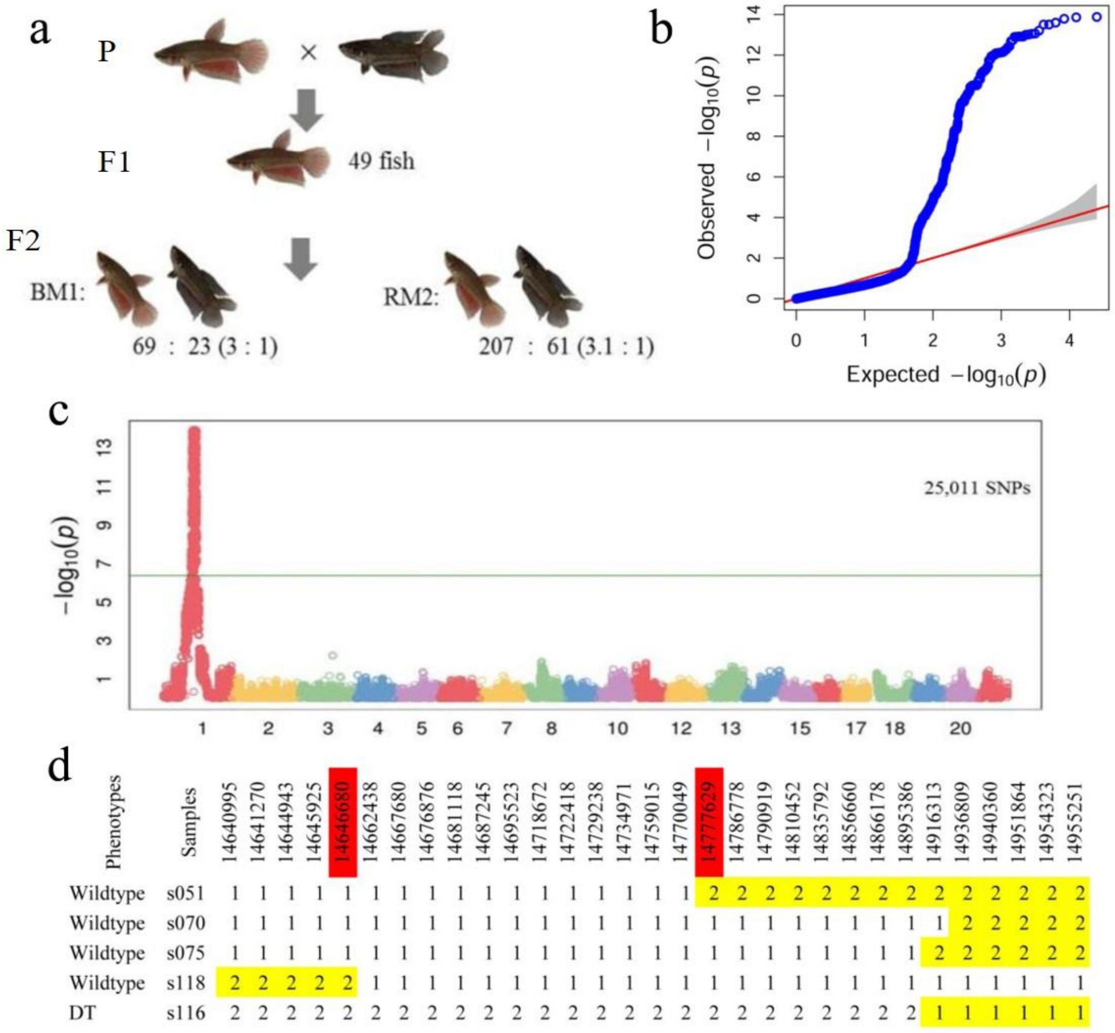

Figure S17. Phenotypic segregation and genome-wide association mapping for finnage variations in fighting fish. (**a**) Phenotypic segregation of single-tail and double-tail fighting fish in two F2 families: BM1 and RM2. The pedigree is set up by crossing homozygous single-tail (F0B1male) and double-tail (DtY2female) P generation fish. All F1 fish are single-tail, while F2 fish show an overall ratio of single-tail to double-tail of 3:1. (**b**) Q-Q plot for *P* values of genome-wide association study under compressed mixed linear model. (**c**) Genome-wide association mapping of ST/st locus using BM1 and RM2 families with the R package GAPIT(Lipka, et al. 2012). Only one significant locus is identified at LG1. Genome-wide significance cut-off value is denoted with green line. (Note, the picture of P and F1 generations here is not the real fish used for setting up these two families, as the fish could not expand their caudal and dorsal fin completely for photography. Photos of DtY2female and F0B1male are shown in **Figure S2**). (**d**) Recombinants around the genetic locus for double-tail mutant, revealed by RAD sequencing-based SNP markers.

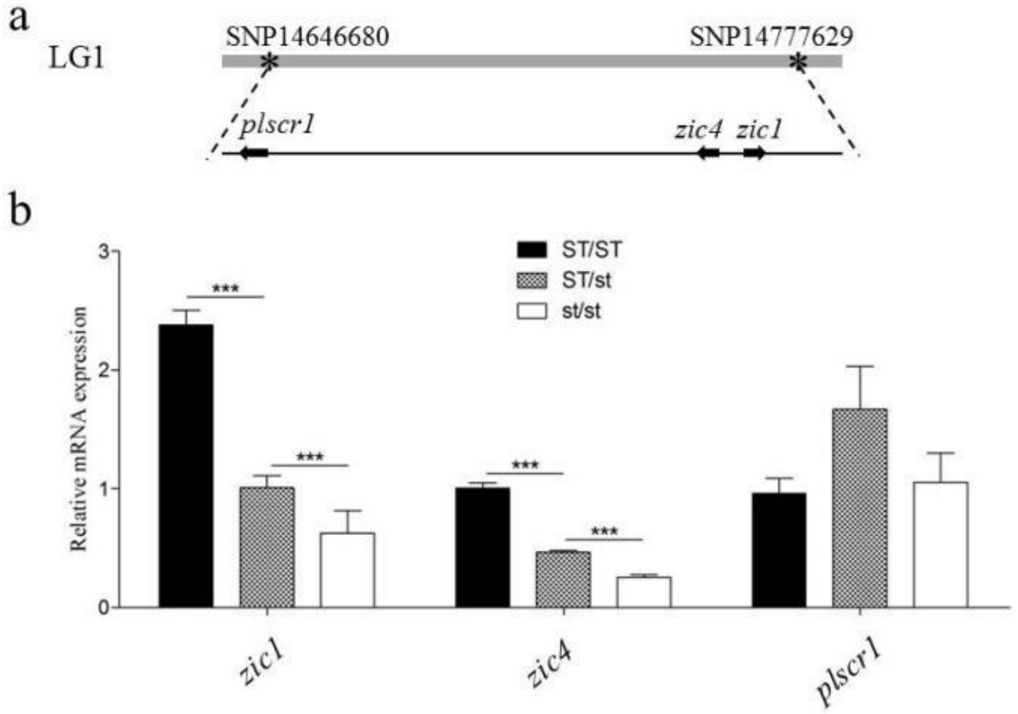

Figure S18. Fine scale mapping for double-tail mutant and expression patterns of candidate genes. (**a**) Genomic region/haplotypes showing no recombination within 502 genotyped individuals and genomic organization of three predicted genes: *zic1*, *zic4* and *plscr1* within this region. (**b**) Expression patterns of *zic1*, *zic4* and *plscr1* in homozygous single-tail, heterozygous single-tail and double-tail fighting fish. The RNA for examination of expression were collected from peduncle and tail of fish. The genotypes are determined by both SNP markers identified in association mapping studies and the Indel marker developed in this study. The differences between groups are examined using Mann-Whitney tests (n =3; *, $P < 0.05$; **, $P < 0.01$; ***, $P < 0.001$).

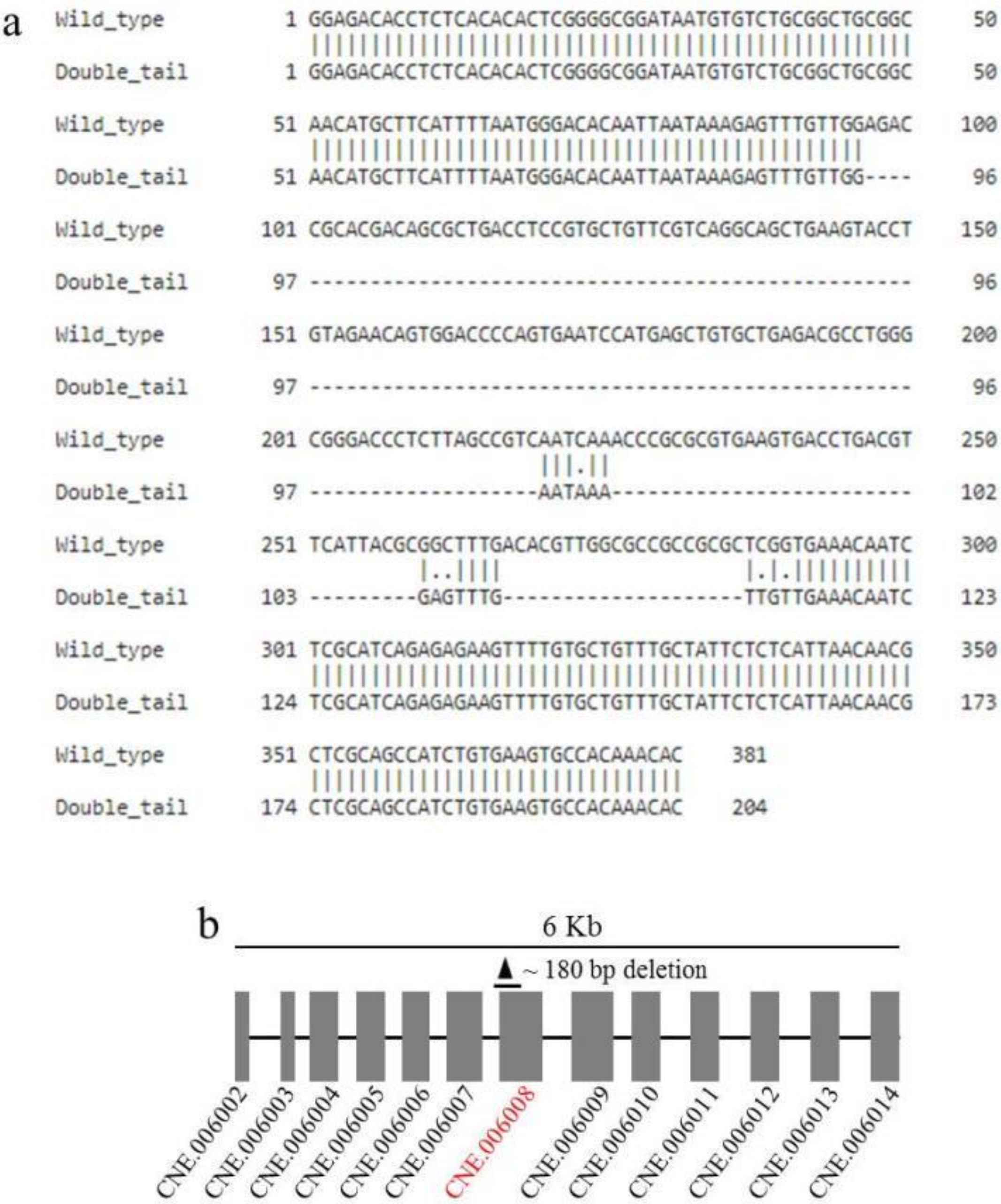

Figure S19. Genomic sequences and organization for conserved non-coding elements around the candidate deletion in double-tail fighting fish. (**a**) Sequence alignment of the candidate deletion (~ 180 bp) and its flanking region between wild-type (single-tail) and double-tail mutant. (**b**) The candidate deletion for double-tail mutant is overlapping with CNE.006008 and is located in a cluster of CNEs with a length of ~ 6 kb containing 13 predicted CNEs.

a

GTCATTTTCCACTGGAGATATTATTCTGTTGTTCTACACTGATTTATTTTTTCACAGCTAAAAGAAG
CTGCAATTACTGGATTGAAGTCTGTTTGTGTGATAAATACTATTCCACTGTGTTGTTTGATAAATAAG
AACATAAGTCAAAGCTTGCACTATGTATAGTAATTAAAGTGAAAAATTCCATCACGGTGTAAATCCTT CNE.006007
AATTGGACTCCCAGAGAGATATTGAAAATTTGTCTTTAGTAATAGGTGATGACTAGGGACAGAAAAAC
AAAGCGTGCTGAAATGTGGCTCTTGTA**GTTCAATTAATCAAACG**CGGAAAACAACCAATTTAGCACAG g296
CTCATTCAGGGAATCACCACTTTCAGGTAATGCAGTTTTAATCATCGCCGCACAAGAGCCAAAAAAAA
AAAAAAAGGA**GACACCTCTCACACACT**CGGGGCGGATAATGTGTCTGCGGCTGC**GGCAACATGCTTCA** g415/gT1
TTTTAATGGGACACAATTAATAAAGAGTTTGTTGGAGACCGCACGACAGCGCTGACCTCCGTGCTGTT Deletion
CGTCAGGCAG**CTGAAGTACCTGTAGAACAG**TGGACCCCAGTGAATCCATGAGCTGTGCTGAGACGCCT gT3
GGGCGGGACCCTCTTAGCCGTCAATCAAACCCGCGCGTGAAGTGACCTGACGTTC**ATTACGCGGCTTT** gT8
GACACGTTGGCGCCGCCGCGCTCGGTGAAACAATCTCGCATCAGAGAGAAGTTTTGTGCTGTTTGCT gT4
ATTCTCTCATTAACAACGCTCGCAGCCA**TCTGTGAAGTGCCACAA**ACACACCTGCTCCAGTGAACGCC g772
TGTAATGGC**TCCTCGCTCTGTGTGCG**TGGCTCTGACTGTCTCAAGGAGAGCGGGGCTTTTATGGACCT g821
GTCATTGCAGGATCATCAACTAGACGCACGGCACCTTGTACGCCACTCGCATAATTATGGATATTGTA
AAGCATTAAAAGTGATCCATTTAGCCGCCTCTGTCACCAGAGGAGTTATTAAGCCACTTTGTGTTGAT CNE.006008
TTCTATGTTCATAATGTGGTGTATTAAAGCACGCCCAACTGTGTGAATTTGGAGCTCTGTTGCAAAAC
AAGGAGTT

b

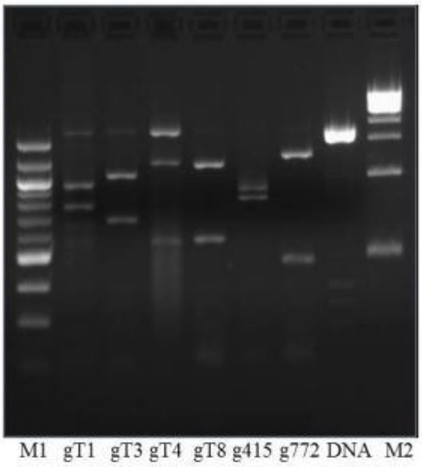

Figure S20. Design of gRNAs and in vitro test of these gRNAs for CRISPR/Cas9 knockout in the target of CNE.006008. (**a**) Sequences of eight gRNAs including g296, g415, gT1, gT3, gT8, gT4, g772 and g821 (underlined) and their corresponding genomic positions between CNE.006007 and CNE.006008. The deletion is indicated with red, while CNEs are shaded with grey. (**b**) In vitro examination of the above gRNAs using Cas9 Nuclease protein (NEB, #M0386M). PCR fragments including CNE.006008 are significantly cleaved by gT3, gT8, g415 and g772, while gT1 and gT4 show partial digestion. However, g296 and g821 show little digestion to the targeted DNA fragments. Based on these results, a series of combinations of g415, g772, gT3 and gT8 are used for injection.

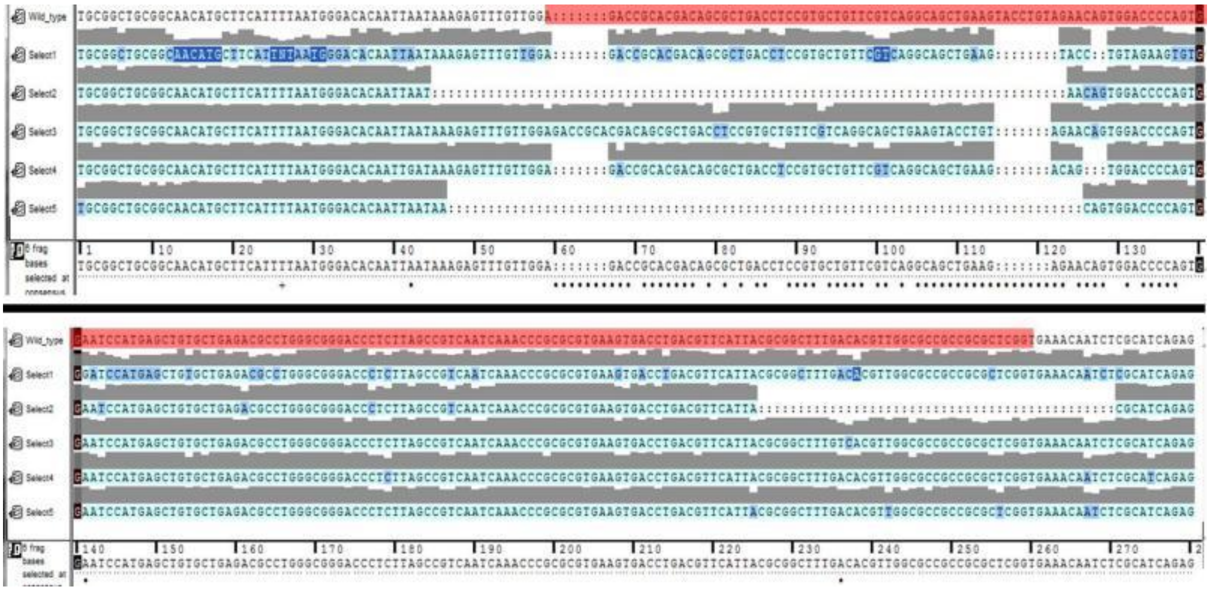

Figure S21. Alignment of wild-type and mutant ST locus, generated by CRISPR/Cas9 system, where the longest deletion we obtained is shown (Select2). In Select2, a total of 125 bp is deleted from the ST allele and ~ 60% of the sequences are overlapping with the ~180-bp deletion of st allele (shaded in red) in double-tail fish.

3 Supplementary tables for the main text

Table S1. Samples, libraries, sequencing reads and depth used for whole-genome sequencing, RNA sequencing and RAD sequencing. All sequences were produced on Illumina Nextseq 500 platform. Fish used for whole-genome sequencing are also shown in **Figure S2**. The major female and male genome assemblies are based on varying sized insert libraries. The genotypes were determined as described in **Figure S2.**

Phenotypes	Genotypes/Description	Library type	Insert size	Raw reads (M)	Reads length (bp)
Yellow single-tail female	Hom. Single-tail, Albino	Paired-end	270 bp	85.7	2X150
(Female assembly)		Paired-end	350 bp	23.5	2X150
		Paired-end	550 bp	24.0	2X150
		Mate-pair	3 kb	32.0	2X150
		Mate-pair	5 kb	32.0	2X150
		Mate-pair	10 kb	48.0	2X150
		Mate-pair	15 kb	40.0	2X150
		Mate-pair	20 kb	4.8	2X150
Albino double-tail male	Double-tail, Albino	Paired-end	275 bp	58.3	2X150
(Male assembly)		Mate-pair	3 kb	44.8	2X150
		Mate-pair	5kb	52.5	2X150
		Mate-pair	10 kb	64.2	2X150
		Mate-pair	15 kb	14.0	2X150
Black single-tail male (F0B1)	Hom. Single-tail, Hom. melanin	Paired-end	550 bp	108.1	2X150
Red double-tail female (DtY2)	Double-tail, Albino	Paired-end	550 bp	90.2	2X150
RNAseq_1	pooled mRNA from individual 1	Paired-end	200 bp	37.1	2X150
RNAseq_2	pooled mRNA from individual 2	Paired-end	200 bp	37.3	2X150
RNAseq_3	pooled mRNA from individual 3	Paired-end	200 bp	35.3	2X150
RNAseq_4	total RNA of one female (non-rRNA)	Paired-end	200 bp	27.3	2X150
RNAseq_5	total RNA of one male (non-rRNA)	Paired-end	200 bp	26.9	2X150
RADseq_1	Four parents of 2 full-sib families	Single-end	500 bp	16.9	1X150
RADseq_2	Two mapping families (366 individuals)	Single-end	500 bp	6.5	1X150
RADseq_3	The other populations (136 individuals)	Single-end	500 bp	5.2	1X150

Table S2. Summary statistics of genome assemblies of four fighting fish. Each fish is denoted with genotypes for tail (S and s for single tail and double tail, respectively) and melanin pigmentation (B and b for melanin-pigmented and albino, respectively). The genotypes were determined according to both cross tests and phenotype-associated DNA markers obtained in the following mapping and association tests. The first two are assembled using ALLPATHS-LG (Gnerre, et al. 2011), the last two using ABYSS2.0 (Jackman, et al. 2017). The sequencing libraries are listed in **Table S1** and Photos are in **Figure S2**

Summary statistics	Yellow single-tail female (SS, bb)	Transparent double-tail male (ss, bb)	F0B1male, Black single-tail male (SS, BB)	DtY2female, Red double-tail female (ss, bb)
No. scaffolds	2871	3252	46399	49029
N50 contigs	21.3 kb	17.3 kb	14.2 kb	13.5 kb
N50 scaffolds	2100 kb	1861 kb	20.5 kb	18.9 kb
Assembly length	424.9 Mb	411.1 Mb	372.7 Mb	370.3 Mb

Table S3. Quality estimation of the yellow single-tail female and albino double-tail male genome assemblies. Assessments were carried out based on *de novo* RADtags- and transcripts-based sequence mapping, and by mapping to Actinopterygii gene set of BUSCO database (Simão, et al. 2015)

	Summary statistics	RADtags	Transcripts	BUSCO
Female	Total loci	518188	71116	4584
	Mapped loci	496511	70823	4475
	Mapping rate	0.958	0.996	0.976
Male	Total loci	518188	71116	4584
	Mapped loci	488349	70563	4480
	Mapping rate	0.942	0.992	0.977

Table S4. Summary statistics of transposable elements (TE) content in the fighting fish genome**.**

TE feature	No. elements	Total length (bp)
DNA	42393	7,901,517 (2.1%)
LINE	27452	9,477,886 (2.5%)
SINE	6743	1,085,301 (0.3%)
LTR	14668	5,084,408 (1.3%)
Others	4308	719,908 (0.2%)
Unknown	30484	7,731,701 (2.0%)
Simple_repeats	198967	8,573,720 (2.3%)
Overall	325015	40,574,441 (10.7%)

Table S5. Summary statistics of two sex-averaged genetic maps, including the number of SNPs and length of each linkage group (LG) for BM1 and RM2 mapping families, respectively. The linkage maps were constructed based on RADtags. A total of 25,263 and 14,082 SNPs were used to construct the 21 linkage groups of the RM2 and BM1 linkage maps, respectively.

	RM2 sex_averaged		BM1 sex_averaged	
LGs	SNPs	cM	SNPs	cM
LG1	1846	66.76	1058	65.79
LG2	1845	59.92	982	58.69
LG3	1702	55.93	937	60.21
LG4	1515	58.85	874	59.98
LG5	1483	51.83	814	58.15
LG6	1389	61.76	786	49.18
LG7	1345	54.53	741	51.15
LG8	1326	50.71	685	54.46
LG9	1238	57.13	686	60.02
LG10	1112	45.41	685	54.34
LG11	1108	53.55	678	51.72
LG12	1090	47.67	624	54.91
LG13	999	50.58	612	49.85
LG14	972	50.87	605	59.54
LG15	945	52.97	604	57.80
LG16	950	46.45	562	53.73
LG17	936	53.67	483	50.72
LG18	927	49.56	459	46.47
LG19	925	51.74	422	59.77
LG20	872	53.20	393	60.99
LG21	738	54.85	392	59.76
Sum	25263	1127.94	14082	1177.23

Table S6. Pearson's correlation coefficient (ρ) between each linkage group of genetic maps and the corresponding physical maps for female and male assemblies based on RM2 and BM1 sex-averaged genetic maps.

Assemblies/	ρ value in female assembly		ρ value in male assembly	
LGs	RM2	BM1	RM2	BM1
LG1	0.929	0.937	0.997	0.995
LG2	0.991	0.988	0.987	0.986
LG3	0.999	0.997	0.997	0.996
LG4	0.991	0.994	0.990	0.971
LG5	0.995	0.979	0.992	0.976
LG6	0.989	0.977	0.990	0.975
LG7	0.987	0.858	0.990	0.872
LG8	0.993	0.994	0.993	0.994
LG9	0.982	0.958	0.991	0.982
LG10	0.996	0.969	0.995	0.969
LG11	0.995	0.984	0.995	0.987
LG12	0.997	0.994	0.997	0.993
LG13	0.990	0.993	0.991	0.994
LG14	0.988	0.971	0.963	0.958
LG15	0.995	0.989	0.995	0.989
LG16	0.992	0.997	0.994	0.996
LG17	0.996	0.985	0.996	0.984
LG18	0.995	0.996	0.996	0.994
LG19	0.998	0.991	0.998	0.990
LG20	0.991	0.990	0.990	0.992
LG21	0.996	0.986	0.999	0.988

Table S7. Genomic sequences anchored onto linkage groups/pseudo-chromosomes for female and male assemblies

LGs	Female assembly length (bp)	Male assembly length (bp)
LG1	32,414,375	31,681,424
LG2	30,483,764	30,799,469
LG3	27,379,829	25,869,756
LG4	19,511,998	17,594,978
LG5	20,628,903	20,002,803
LG6	20,412,404	19,450,542
LG7	20,539,272	20,677,044
LG8	19,278,563	19,001,869
LG9	15,181,724	15,410,798
LG10	17,544,266	16,731,250
LG11	14,845,826	14,540,785
LG12	19,281,159	19,147,109
LG13	18,550,546	17,242,247
LG14	17,668,581	17,889,003
LG15	15,625,086	15,429,903
LG16	13,445,423	14,054,825
LG17	12,742,746	11,869,953
LG18	20,725,612	19,525,911
LG19	15,076,098	14,331,182
LG20	16,727,899	16,478,664
LG21	15,359,059	14,089,977
Sum	403,423,133 (94.9 %)	391,732,092 (95.3%)
Oriented	296,452,614 (69.8 %)	277,095,002 (67.4%)

Table S8. Samples used for inferring signature of selection and mapping for elephant ear locus, including major color and fin phenotypes and sequencing depth. Domesticated samples 12, 18, 25, 26 and 27 are from Fan et al. (2018) (Fan, et al. 2018).

Samples (origin)	Major body color	Major Fin shape	Sequence coverage
Elephant_ear_01	M/U	Elephant ear (EE)	~ 15×
Elephant_ear_02	M/U	Elephant ear (EE)	~ 12×
Elephant_ear_03	M/U	Elephant ear (EE)	~ 8×
Elephant_ear_04	M/U	Elephant ear (EE)	~ 10×
Elephant_ear_05	M/U	Elephant ear (EE)	~ 10×
Elephant_ear_06	M/U	Elephant ear (EE)	~ 10×
Elephant_ear_07	M/U	Elephant ear (EE)	~ 14×
Elephant_ear_08	M/U	Elephant ear (EE)	~ 16×
Elephant_ear_09	M/U	Elephant ear (EE)	~ 20×
Domesticated_01	Yellow	Plakat (PK)	~ 30×
Domesticated_02	Red	Horsetail (HT)	~ 15×
Domesticated_03	M/U	Plakat (PK)	~ 30×
Domesticated_04	Red	Halfmoon (HM)	~ 21×
Domesticated_05	Red	Plakat (PK)	~ 18×
Domesticated_06	Yellow	Plakat (PK)	~ 14×
Domesticated_07	Red	Horsetail (HT)	~ 10×
Domesticated_08	Yellow	Plakat (PK)	~ 8×
Domesticated_09	Transparent	Doubletail (DT)	~ 30×
Domesticated_10	Black	Veiltail (VT)	~ 30×
Domesticated_11	Transparent	Doubletail (DT)	~ 30×
Domesticated_12	Black	Veiltail (VT)	~ 30×
Domesticated_13	Black	Veiltail (VT)	~ 30×
Domesticated_14	Red	Veiltail (VT)	~ 10×
Domesticated_15	M/U	Veiltail (VT)	~ 8×
Domesticated_16	M/U	Halfmoon (HM)	~ 30×
Domesticated_17	M/U	Plakat (PK)	~ 12×
Domesticated_18	M/U	Plakat (PK)	~ 10×
Domesticated_19	White	Halfmoon (HM)	~ 25×
Domesticated_20	Red	Halfmoon (HM)	~ 18×
Domesticated_21	White	Veiltail (VT)	~ 14×
Domesticated_22	Blue	Halfmoon (HM)	~ 15×
Domesticated_23	White	Halfmoon (HM)	~ 12×
Domesticated_24	Blue	Doubletail (DT)	~ 10×
Domesticated_25	M/U	Halfmoon (HM)	~ 30×
Domesticated_26	M/U	Halfmoon (HM)	~ 30×
Domesticated_27	Blue	Plakat (PK)	~ 20×
Domesticated_28	Blue	Plakat (PK)	~ 10×
Wild_01 (Thailand, Samutsakorn)	N.A.	N.A.	~ 25×
Wild_02 (Thailand, Samutsakorn)	N.A.	N.A.	~ 25×
Wild_03 (Thailand, Samutsakorn)	N.A.	N.A.	~ 26×
Wild_04 (Cambodia, Cambodia River)	N.A.	N.A.	~ 25×
Wild_05 (Cambodia, Cambodia River)	N.A.	N.A.	~ 23×
Wild_06 (Thailand, Samutsakorn)	N.A.	N.A.	~ 24×

Table S9. Summary statistics of wild-type melanin cell pigmented and albino fish and their corresponding genotypes at the locus of 366bp deletion, 25 kb upstream of the *mitfa* gene. Within these genotyped fish, 91 were randomly collected from several Asian countries, including China, Thailand, Malaysia, Singapore and Indonesia, while the others from the same pedigree.

Phenotypes	Number of fish	Genotypes
Wild type	288	BB
Wild type	471	Bb
Albino	223	bb

Table S10. Summary statistics of double tail and single tail fish and their corresponding genotypes at the ~ 180bp deletion locus within CNE.006008. Within these genotyped fish, 91 were randomly collected from several Asian countries, including China, Thailand, Malaysia, Singapore and Indonesia, while the others from the same pedigree.

Phenotypes	Number of fish	Genotypes
Single tail	468	ST/ST
Single tail	378	ST/st
Double tail	186	st/st

Table S11. The number of embryos of fighting fish injected with zebrafish enhancer detection vector (ZED) (Bessa, et al. 2009) constructs to study the enhancer activities of conserved non-coding elements (CNEs). The number of survived embryos showing consistent GFP expression and the total number of survived embryos showing expression of internal control RFP, at the time of recoding are denoted.

ZED constructs	GFP/Total number	Feature
CNE.006008_ST	9/43	Downstream of *Zic1*/*Zic4*
CNE.006008_st	0/50	Downstream of *Zic1*/*Zic4*

Table S12. Primers used in this study**.**

Primer name	Sequences: 5'-3'	Annotation
Betta_geo_F3	CTCAAATCCGCCAATATGCT	Genome estimation qPCR 1
Betta_geo_R3	TGCTCGCTTGTTATCCTCCT	Genome estimation qPCR 1
Betta_geo_F4	TATGCTCACAGCGTTCGTTC	Genome estimation qPCR 2
Betta_geo_R4	GTCTGATGCTCCCCATGACT	Genome estimation qPCR 2
RT_b_actin_F	GGGACGACATGGAGAAGATC	Reverse Transcription PCR
RT_b_actin_R	GCAGTGGTGGTGAAGCTGTA	Reverse Transcription PCR
EF1aF1	CCGCTCAGGTGATCATTCTT	QPCR reference Ef1a
EF1aF2	CGTAACCACCGAGGTGAAGT	QPCR reference Ef1a
Betta_ActinF3	CGGTCGTACCACAGGTATCG	QPCR reference Actin
Betta_ActinR3	AGTGGTGGTGAAGCTGTAGC	QPCR reference Actin
14939910F	GTTGGACTCAGCACTGACCA	Fine mapping ST/st
14939910R	CCTGTGCCACTCTTTCTTCC	Fine mapping ST/st
14894936F	ACAGGGCCTGCAAGATAATG	Fine mapping ST/st
14894936R	CTCCGGGTCGCAATTACTTA	Fine mapping ST/st
14844429F	GTGACAGGCACTGTGGCTAA	Fine mapping ST/st
14844429R	TGAATTGGGAAGTCAGTCCA	Fine mapping ST/st
14809773F	CCTGCAACAAAGTGAGCAAA	Fine mapping ST/st
14809773R	TGTCGCTGCCTCTTACAGTG	Fine mapping ST/st
14769599F	TCAAGTTGTTGCAGCTGGTC	Fine mapping ST/st
14769599R	CTCAATAACAACCCGGTGCT	Fine mapping ST/st
14644493F	TTGAGTGCAGCTTCCTGCTA	Fine mapping ST/st
14644493R	GCAAGTCTCTGGGGTGACAT	Fine mapping ST/st
ZED/pGL3-Promoter_F	CCATGTGTTTTCTGCAATGG	ZED vector construction related to CNE.006008
ZED/pGL3-Promoter_R	CCTCCCCATCATTACACAGG	ZED vector construction related to CNE.006008
Zic_enhancer_gRNA_T1	TAATACGACTCACTATAGGGGCAACATGCTTCATTTTAAGTTTTAGAGCTAGAAATAGC	gRNA for knockout CNE.006008
Zic_enhancer_gRNA_T3	TAATACGACTCACTATAGGCTGAAGTACCTGTAGAACAGGTTTTAGAGCTAGAAATAGC	gRNA for knockout CNE.006008
Zic_enhancer_gRNA_T8	TAATACGACTCACTATAGGATTACGCGGCTTTGACACGTGTTTTAGAGCTAGAAATAGC	gRNA for knockout CNE.006008
Zic_enhancer_gRNA_T4	TAATACGACTCACTATAGGCACGTTGGCGCCGCCGCGCTGTTTTAGAGCTAGAAATAGC	gRNA for knockout CNE.006008
Zic_enhancer_gRNA_296	TAATACGACTCACTATAGGAGTTCAATTAATCAAACGGTTTTAGAGCTAGAAATAGC	gRNA for knockout CNE.006008
Zic_enhancer_gRNA_415	TAATACGACTCACTATAGGAGACACCTCTCACACACTGTTTTAGAGCTAGAAATAGC	gRNA for knockout CNE.006008
Zic_enhancer_gRNA_772	TAATACGACTCACTATAGGTTTGTGGCACTTCACAGAGTTTTAGAGCTAGAAATAGC	gRNA for knockout CNE.006008
Zic_enhancer_gRNA_821	TAATACGACTCACTATAGGCTCCTCGCTCTGTGTGCGGTTTTAGAGCTAGAAATAGC	gRNA for knockout CNE.006008
Universal oligos for gRNAs	AAAAGCACCGACTCGGTGCCACTTTTTCAAGTTGATAACGGACTAGCCTTATTTTAACTTGCTATTTCTAGCTCTAAAAC	Universal oligos for synthesizing gRNA templates
Zic4_down_PFF	AATCACCACTTTCAGGTAATGC	Test gRNA/genotyping by Sanger sequencing
Zic4_down_PR2	CCTGCAATGACAGGTCCATA	Test gRNA/genotyping by Sanger sequencing
Zic4_Q1F	ACTGGTGAGAAGCCCTTCAA	QPCR for zic4
Zic4_Q1R	CTTGCAGTGCACCTTCATGT	QPCR for zic4

Zic1_Q1F	ACGAATCCCAAAAAGTCGTG	QPCR for zic1
Zic1_Q1R	ACTTGTCGCACATTTTGCAG	QPCR for zic1
Plscr1_QF	AACACGGTGGGGTACATCAT	QPCR for plscr1
Plscr1_QR	AACTGGATGCCGAAGTTGTC	QPCR for plscr1
Zic_locus_F1	TGCCTGAGAATTAGCACGTC	Genomic region amplification of zic1/zic4 locus (long PCR)
Zic_locus_R1	TCTCTATTCAGTATCAAAGCGTCCT	Genomic region amplification of zic1/zic4 locus (long PCR)
Zic_locus_F2	GCAGGACGCTTTGATACTGAAT	Genomic region amplification of zic1/zic4 locus (long PCR)
Zic_locus_R2	AGGAAGAATATCTGTGCGTGGT	Genomic region amplification of zic1/zic4 locus (long PCR)
Zic_locus_F3	ATAGAGAGAGGGGGAGGAAGAATAT	Genomic region amplification of zic1/zic4 locus (long PCR)
Zic_locus_R3	TCCCAACTTGGCGAAATCT	Genomic region amplification of zic1/zic4 locus (long PCR)
RT_Camkv_F	CTCCGTACTGGGACGACATT	RT-PCR for camkv
RT_Camkv_R	TTTGCAGGGAATGGAGGTAG	RT-PCR for camkv
RT_Sema3f_F	CGGAGGAGAGACATGAGGAG	RT-PCR for sema3ff
RT_Sema3f_R	AGCTCATTCCGAACAGTCGT	RT-PCR for sema3ff
RT_10705_F	TGCAGGGAAGAAACAGAAGG	RT-PCR for 10705
RT_10705_R	TCTTGAACGAGACGCAAAGA	RT-PCR for 10705
RT_10706_F	AGATCTACCCAACGCACTCG	RT-PCR for 10706
RT_10706_R	GGACCTGAACCAGTCCAAAA	RT-PCR for 10706
RT_Naaa_F	AGTGGTGGAACTGGTGGAAG	RT-PCR for naaa
RT_Naaa_R	AACGAACCTCTCCAGGGACT	RT-PCR for naaa
RT_Bhlhe40_F	TGATCAGCCACATCCAAAAA	RT-PCR for bhlhe40
RT_Bhlhe40_R	GTAGAAGGGGAGGCAAAAGG	RT-PCR for bhlhe40
RT_Itpr1_F	TTGTCATCGTCTTGCCTCAG	RT-PCR for itpr1
RT_Itpr1_R	TCACACGTTCGCTCTTTGTC	RT-PCR for itpr1
RT_Setmar_F	CCGCCGAGTTTCATACATTT	RT-PCR for setmar
RT_Setmar_R	AATGCTCCCTTATGGCAATG	RT-PCR for setmar
RT_Lrrn1_F	TTGGACATTGGCATGAGTGT	RT-PCR for lrrn1
RT_Lrrn1_R	TGCTGGTTGTAAATGGGTGA	RT-PCR for lrrn1
RT_10715_F	CCTTTCCCAACATGGCTAGA	RT-PCR for 10715
RT_10715_R	CGGATGGAGAAGAAGACGAG	RT-PCR for 10715
RT_Oxtr_F	GCAACCTCACCGTGGATAGT	RT-PCR for oxtr
RT_Oxtr_R	ACCTCGACCCTGGAGAAGAT	RT-PCR for oxtr
RT_Trnt1_F	TCACCACTGACTGGCAGAAG	RT-PCR for trnt1
RT_Trnt1_R	GTCGGTGAGCAGACACTGAA	RT-PCR for trnt1
RT_Arl6ip5_F	GTCAGCAGGAAGAGGGTCTG	RT-PCR for arl6ip5
RT_Arl6ip5_R	TAGAAACCTGGCAGCGACTT	RT-PCR for arl6ip5
RT_Frmd4b_F	GGTTCAGCCCAAGTTGTTGT	RT-PCR for frmd4b
RT_Frmd4b_R	AAAAACCGGGAGCTCCTTTA	RT-PCR for frmd4b
RT_Mitfa_1F	TGGGTGGTAAAACAGGAAGC	RT-PCR for mitfa
RT_Mitfa_1R	AAATCCCTCTGGCCTTTGAT	RT-PCR for mitfa
RT_Dhqs_F	TGATCAGCCCCATTACCTTC	RT-PCR for dhqs
RT_Dhqs_R	CTGATGTAAGGGGTGCGTCT	RT-PCR for dhqs
RT_10722_F	ACATGAGTCAGGCCAAGAGG	RT-PCR for 10722

RT_10722_R	CTTTCCTGCGTGGTCATTTT	RT-PCR for 10722
RT_Foxp1_F	CTGTCCCAGACCCACTCTGT	RT-PCR for foxp1
RT_Foxp1_R	GTTGTTGCTGCTGCTGTTGT	RT-PCR for foxp1
Q_Sema3f_QF	AACCCCACATAAATCCTCTCAA	QPCR for sema3f
Q_Sema3f_QR	AGCTCATTCCGAACAGTCGT	QPCR for sema3f
Q_Itpr1_QF	GTGGCCAAGACACAGACTGA	QPCR for ttpr1
Q_Itpr1_QR	ACCTCCTGCCTCTGACTGAA	QPCR for ttpr1
Q_Setmar_QF	GCTAACCGTGTCGTCCAGAT	QPCR for setmar
Q_Setmar_QR	AATGCTCCCTTATGGCAATG	QPCR for setmar
Q_Lrrn1_QF	CAGCGCACAGCTTATGAAAA	QPCR for lrrn1
Q_Lrrn1_QR	TGCTGGTTGTAAATGGGTGA	QPCR for lrrn1
Q_Frmd4b_QF	TCTAAGACCTTGGGGCCTTT	QPCR for frmd4b
Q_Frmd4b_QR	AAAAACCGGGAGCTCCTTTA	QPCR for frmd4b
Q_Mitfa_Q1F	TGGACTAATGGACCCAGCTC	QPCR for mitfa
Q_Mitfa_Q1R	AAATCCCTCTGGCCTTTGAT	QPCR for mitfa
Q_Dhqs_QF	TGATCAGCCCCATTACCTTC	QPCR for dhqs
Q_Dhqs_QR	TCTTGGCCCCATAGATTTTG	QPCR for dhqs
Q_Mitfb_QF	AAGCGTCGGTGGACTACATC	QPCR for mitfb
Q_Mitfb_QR	TGAGGATGTCGTTGAGCTTG	QPCR for mitfb
Mitfa_E2_gRNA	TAATACGACTCACTATAGGGGTGGCATACCGTGCTCCGGGTTTTAGAGCTAGAAATAGC	gRNA for knockout mitfa
Mitfa_E3_gRNA	TAATACGACTCACTATAGGGAGCTGGGTCCATTAGTCCAGTTTTAGAGCTAGAAATAGC	gRNA for knockout mitfa
Mitfa_E4_gRNA	TAATACGACTCACTATAGGGACCTGGGATTGGAAGTCCCGTTTTAGAGCTAGAAATAGC	gRNA for knockout mitfa
Mitfa_E5_gRNA	TAATACGACTCACTATAGGCTGGACAAGCCTGGGTCCAGGTTTTAGAGCTAGAAATAGC	gRNA for knockout mitfa
Mitfa_E2-5_F	TGGAATGATGTCGGATTTTT	PCR for DNA fragment test gRNA for mitfa
Mitfa_E2-5_R	CCAACAGGTGTGAAGCCTTA	PCR for DNA fragment test gRNA for mitfa
Mitfa_E3_sequencing_F	ATACAGATGGATGATGTCATCG	Genotyping mitfa knockout by Sanger sequencing and PCR for T7 Endonuclease assays
Mitfa_E3_sequencing_R	CTGTATACGAAGCATTAGAT	Genotyping mitfa knockout by Sanger sequencing and PCR for T7 Endonuclease assays
Mitfa_366bp_deletion_GTF	AGCAGCAGAGGCCATCTTTA	Genotyping 366-bp deletion upstream of mitfa
Mitfa_366bp_deletion_GTR	TGCAAGAAGAACAGCGTGTC	Genotyping 366-bp deletion upstream of mitfa
Kcnh8_QF	GCAAGGAGTTCAAGGACGAG	Kcnh8 qPCR
Kcnh8_QR	TTGGATGGCTTTGGCTTTAG	Kcnh8 qPCR
Fkbp14_QF	CGAGGGCTACTTCGAAAATG	Fkbp14 qPCR
Fkbp14_QR	CTGGAGGTACGACCAGCTTC	Fkbp14 qPCR
Evx1_QF	TCAAGCCTGACACTGACCTG	Evx1 qPCR
Evx1_QR	GTGTCCGTTAGTGGGGAGAA	Evx1 qPCR
Col8a2_QF	GCAAGGAGTGAGGGGTGATA	Col8a2 qPCR
Col8a2_QR	GCCTCTCCTGCAACATCTTC	Col8a2 qPCR
Col16a1_QF	TTGATGGACAACCAGGACAA	Col16a1 qPCR
Col16a1_QR	ACCATTAAGACCGGGGATTC	Col16a1 qPCR
Col9a2_QF	ACTACCCCACGGCTAAAGGT	Col9a2 qPCR
Col9a2_QR	CCCGGTAAAATTCCTTCTCC	Col9a2 qPCR

4 References

Bateman A, Coin L, Durbin R, Finn RD, Hollich V, Griffiths-Jones S, Khanna A, Marshall M, Moxon S, Sonnhammer EL. 2004. The Pfam protein families database. *Nucleic Acids Research* 32:D138-D141.

Bessa J, Tena JJ, de la Calle-Mustienes E, Fernández-Miñán A, Naranjo S, Fernández A, Montoliu L, Akalin A, Lenhard B, Casares F. 2009. Zebrafish enhancer detection (ZED) vector: a new tool to facilitate transgenesis and the functional analysis of cis-regulatory regions in zebrafish. *Developmental Dynamics* 238:2409-2417.

Catchen J, Hohenlohe PA, Bassham S, Amores A, Cresko WA. 2013. Stacks: an analysis tool set for population genomics. *Molecular Ecology* 22:3124-3140.

Fan G, Chan J, Ma K, Yang B, Zhang H, Yang X, Shi C, Chun-Hin Law H, Ren Z, Xu Q. 2018. Chromosome-level reference genome of the Siamese fighting fish Betta splendens, a model species for the study of aggression. *GigaScience* 7:giy087.

Gnerre S, MacCallum I, Przybylski D, Ribeiro FJ, Burton JN, Walker BJ, Sharpe T, Hall G, Shea TP, Sykes S. 2011. High-quality draft assemblies of mammalian genomes from massively parallel sequence data. *Proceedings of the National Academy of Sciences, USA* 108:1513-1518.

Jackman SD, Vandervalk BP, Mohamadi H, Chu J, Yeo S, Hammond SA, Jahesh G, Khan H, Coombe L, Warren RL. 2017. ABySS 2.0: resource-efficient assembly of large genomes using a Bloom filter. *Genome Research* 27:768-777.

Lipka AE, Tian F, Wang Q, Peiffer J, Li M, Bradbury PJ, Gore MA, Buckler ES, Zhang Z. 2012. GAPIT: genome association and prediction integrated tool. *Bioinformatics* 28:2397-2399.

Lucas GA. 1968. A study of variation in the Siamese Fighting Fish, Betta splendens, with emphasis on color mutants and the problem of sex determination. [Iowa State University.

McLeod MJ. 1980. Differential staining of cartilage and bone in whole mouse fetuses by alcian blue and alizarin red S. *Teratology* 22:299-301.

Monvises A, Nuangsaeng B, Sriwattanarothai N, Panijpan B. 2009. The Siamese fighting fish: well-known generally but little-known scientifically. *ScienceAsia* 35:8-16.

Rüber L, Britz R, Tan HH, Ng PK, Zardoya R. 2004. Evolution of mouthbrooding and life-history correlates in the fighting fish genus Betta. *Evolution* 58:799-813.

Simão FA, Waterhouse RM, Ioannidis P, Kriventseva EV, Zdobnov EM. 2015. BUSCO: assessing genome assembly and annotation completeness with single-copy orthologs. *Bioinformatics* 31:3210-3212.

Simpson M. 1968. The display of the Siamese fighting fish, Betta splendens. *Animal Behaviour Monographs* 1:1-73.

Tang H, Zhang X, Miao C, Zhang J, Ming R, Schnable JC, Schnable PS, Lyons E, Lu J. 2015. ALLMAPS: robust scaffold ordering based on multiple maps. *Genome Biology* 16:3.

Wilhelm J, Pingoud A, Hahn M. 2003. Real-time PCR-based method for the estimation of genome sizes. *Nucleic Acids Research* 31:e56-e56.
